# Associations of prenatal exposure to maternal autoimmune disorders with a wide spectrum of psychiatric and neurodevelopmental disorders in offspring—a nationwide cohort study

**DOI:** 10.1093/hropen/hoag026

**Published:** 2026-04-26

**Authors:** Elin Skott, Wenjie Cai, Miranda Stiernborg, Anna Fogdell-Hahn, Maibritt Giacobini, Mika Gissler, Samson Nivins, Catharina Lavebratt

**Affiliations:** Department of Molecular Medicine and Surgery, Karolinska Institutet, Stockholm, Sweden; Center for Molecular Medicine, Karolinska University Hospital Solna, Stockholm, Sweden; Department of Molecular Medicine and Surgery, Karolinska Institutet, Stockholm, Sweden; Center for Molecular Medicine, Karolinska University Hospital Solna, Stockholm, Sweden; Department of Molecular Medicine and Surgery, Karolinska Institutet, Stockholm, Sweden; Center for Molecular Medicine, Karolinska University Hospital Solna, Stockholm, Sweden; Infectious Disease and Microbiome Program, Broad Institute, Cambridge, MA, USA; Department of Women’s and Children’s Health, Uppsala University, Uppsala, Sweden; Department of Molecular Medicine and Surgery, Karolinska Institutet, Stockholm, Sweden; Prima Child and Adult Psychiatry Stockholm AB, Stockholm, Sweden; Department of Molecular Medicine and Surgery, Karolinska Institutet, Stockholm, Sweden; Center for Molecular Medicine, Karolinska University Hospital Solna, Stockholm, Sweden; Department of Data and Analytics, Finnish Institute for Health and Welfare, Helsinki, Finland; Region Stockholm, Academic Primary Health Care Centre, Stockholm, Sweden; Department of Women’s and Children’s Health, Karolinska Institutet, Stockholm, Sweden; Department of Molecular Medicine and Surgery, Karolinska Institutet, Stockholm, Sweden; Center for Molecular Medicine, Karolinska University Hospital Solna, Stockholm, Sweden

**Keywords:** autoinflammatory, pregnancy, type 1 diabetes, pernicious anemia, mental disorder

## Abstract

**STUDY QUESTION:**

Are children exposed *in utero* to various maternal autoimmune disorders (ADs) and autoinflammatory disorders (AIDs) at greater risk for developing any of a wide range of psychiatric or neurodevelopmental disorders?

**SUMMARY ANSWER:**

Prenatal exposure to maternal ADs and AIDs, but for those of the nervous system, was at modest effect sizes associated with a number of primarily early onset neurodevelopmental disorders.

**WHAT IS KNOWN ALREADY:**

Prenatal exposure to maternal immune activation and certain ADs or AIDs has been associated with psychiatric and neurodevelopmental disorders in offspring. However, most studies examined only one specific maternal exposure, such as rheumatoid arthritis, in relation to one specific outcome, such as autism spectrum disorder (ASD). The potential impact of a broader range of maternal AD/AIDs on various mental outcomes remains largely unexplored.

**STUDY DESIGN, SIZE, DURATION:**

A nationwide, population-based cohort study of all live births in Finland from 1996 to 2014, with follow-up to 2021 (N = 1 107 802). Analyses were conducted from October 2023 to December 2025.

**PARTICIPANTS/MATERIALS, SETTING, METHODS:**

Maternal diagnoses of AD/AIDs, recorded prior to and/or during pregnancy, were identified through Finnish National Health Registers (N = 34 033). Cox regression was used to estimate the hazard ratios (HRs) with 99% and 95% CIs of psychiatric and neurodevelopmental disorder diagnoses in offspring to AD/AIDs mothers.

**MAIN RESULTS AND THE ROLE OF CHANCE:**

Of the 1 107 802 births, 3.2% were to mothers with an AD/AID. Offspring exposed to maternal AD/AIDs had a 15% higher risk of a major psychiatric disorder (HR, 1.15 [99% CI, 1.09–1.21]) and an 18% higher risk of a neurodevelopmental disorder (HR, 1.18 [99% CI, 1.14–1.22]).

Combining AD/AIDs according to the main body system affected, associations with offspring’s mental diagnoses were detected mainly for connective tissue AD/AIDs and endocrine AD/AIDs. Although most effect sizes were modest (HRs below 2), there were two notable exceptions with more than 2-fold risk: (i) ASD in autoimmune thyroiditis, and (ii) other behavioral or emotional disorders (F98) in pernicious anemia. A 70–99% higher risk for certain neurodevelopmental disorders or milder regulatory disturbances was observed in offspring exposed to systemic vasculitis, type 1 diabetes mellitus (T1DM), acute thyroiditis, Crohn’s disease, and SIC (being polymyalgia rheumatica, Sjögren’s syndrome, and hypermobility syndrome). The T1DM associations were not completely mediated by adverse birth factors.

**LIMITATIONS, REASONS FOR CAUTION:**

The number of exposure-discordant siblings was insufficient to fully adjust for shared unmeasured familial confounding, and paternal information and breastfeeding data were unavailable. Second, some AD/AIDs were rare, limiting the statistical power. The numbers of exposed cases with outcome in the two associations with largest effect size was limited (n = 9 and 20). Third, rare prodromal AD/AID symptoms cannot be excluded, potentially causing misclassification. Fourth, some AD/AIDs recorded prior to pregnancy might have resolved before pregnancy, unknown to the study. Fifth, grouping AD/AIDs by the main affected body system implies a risk for not detecting true associations of individual AD/AIDs. Finally, the study is exploratory, and the observational design prevents causal inference.

**WIDER IMPLICATIONS OF THE FINDINGS:**

The findings may provide information for maternity care and family planning clinics potentially alleviating concerns among mothers with an AD/AID regarding offspring risk of neurodevelopmental and psychiatric disorder.

**STUDY FUNDING/COMPETING INTEREST(S):**

This research was funded by Prima Child and Adult Psychiatry Stockholm AB (E.S.), the Swedish Research Council, Sweden (C.L., 2022-01188), the Swedish Brain Foundation, Sweden (C.L., FO2024-0194 and FO2025-0276-HK-192), Bo and Ulla Lundevall, Ulf Lundahl Memorial Fund, the regional agreement on medical training and clinical research (ALF) between Region Stockholm and Karolinska Institutet, Sweden (C.L., RS2021-0855 and 2023-0859) and China Scholarship Council (W.C.). The authors have no conflict to declare.

**TRIAL REGISTRATION NUMBER:**

N/A.

WHAT DOES THIS MEAN FOR PATIENTS? The immune system of the pregnant woman can influence the brain development of the fetus. In persons with an autoimmune or autoinflammatory disorder, certain parts of the immune system are sometimes a bit more active. There are many different autoimmune or autoinflammatory disorders, and most are long-term.

This study looked at whether the children to pregnant women with such a disorder more often get a diagnosis of a neurodevelopmental or psychiatric disorder during their childhood or early adulthood. To study this, data from 1.1 million births from nationwide registers in Finland were used.

The researchers found that generally there seemed to be no, or only a little larger risk for neurodevelopmental or psychiatric disorders among children born to mothers with an autoimmune or autoinflammatory disorder, with certain exceptions for some rare maternal disorders. Maternal disorders affecting primarily the brain, e.g. multiple sclerosis, did not imply a higher risk of mental disorder for the born children.

The researchers hope that these findings can be confirmed in another large study.

## Introduction

Autoimmune (AD) and autoinflammatory disorders (AID) are characterized by immune responses against self with the secretion of tissue-damaging cytokines, and ADs also having synthesis of organ-specific or systemic autoantibodies (Wang *et al.*, 2015). The prevalence of ADs is increasing, with women being more affected than men (Conrad *et al.*, 2023). Animal studies show that maternal immune activation (MIA) can disrupt neurotransmitters, leading to behavioral changes in offspring (Quagliato *et al.*, 2021). Human studies suggest an association between MIA and offspring mental health but often focus on specific maternal ADs, and do not fully account for perinatal risk factors or timing of the maternal diagnosis (i.e. before or after pregnancy) (Vinet *et al.*, 2015; Wu *et al.*, 2015; Nielsen *et al.*, 2017; Mataix-Cols *et al.*, 2018; Rom *et al.*, 2018; Tsai *et al.*, 2018; Spann *et al.*, 2019; Chen *et al.*, 2021). Some studies consider temporality (i.e. exposure preceding outcome), but findings remain inconsistent. For example, one study reported an association between maternal rheumatoid arthritis (RA) and autism spectrum disorder (ASD) only when RA was diagnosed after pregnancy (Sun *et al.*, 2022), whereas another study found an ASD association only when RA was diagnosed prior to pregnancy (Yin *et al.*, 2023). Some studies report association for both paternal and maternal AD/AID exposure on offspring neurodevelopment, with a stronger association on the maternal side (Ellul *et al.*, 2022). Importantly, most studies have focused only on offspring neurodevelopmental disorders (NDDs), e.g. ASD, and not other mental disorders.

A Danish nationwide cohort study found that maternal pre-pregnancy onset of type 1 diabetes mellitus (T1DM), RA, and systemic lupus erythematosus (SLE) was associated with a higher offspring risk of mental disorders overall (He *et al.*, 2022). However, associations between specific maternal AD/AID-exposures, i.e. isolated diagnoses or grouped according to the targeted body system, and specific mental disorders in children remain unexplored.

By studying several AD/AIDs within a single cohort, we can compare the effect sizes of associations across different exposures and outcomes. Therefore, our study aimed to explore the associations between maternal AD/AID active during pregnancy and a broad range of individual mental disorders in children exposed during pregnancy. Where relevant, we explored whether these associations were dependent on delivery route or preterm birth (PTB).

## Materials and methods

### Study population and data sources

This nationwide registry-based cohort study included all live births in Finland between January 1996 and December 2014, identified through the Medical Birth Register (MBR) and the Finnish Care Registers for Health Care (HILMO) (N = 1 107 802, Supplementary Table S1). Offspring were followed up until December 2021 using data from HILMO and the Finnish Register on Reimbursed Drugs (RRD). MBR and HILMO are maintained by the Finnish Institute for Health and Welfare. RRD is maintained by the Social Insurance Institution of Finland. The study was approved by data protection authorities in Finland (THL/3922/14.06.00/2022; THL/2722/14.06.00/2023; THL/5391/14.02.00/2022) and Sweden (2023-03041). Since this was strictly register-based, informed consent was not required. Reporting followed the Strengthening the Reporting of Observational Studies in Epidemiology (STROBE) guidelines.

### Exposures

**Figure 1.**
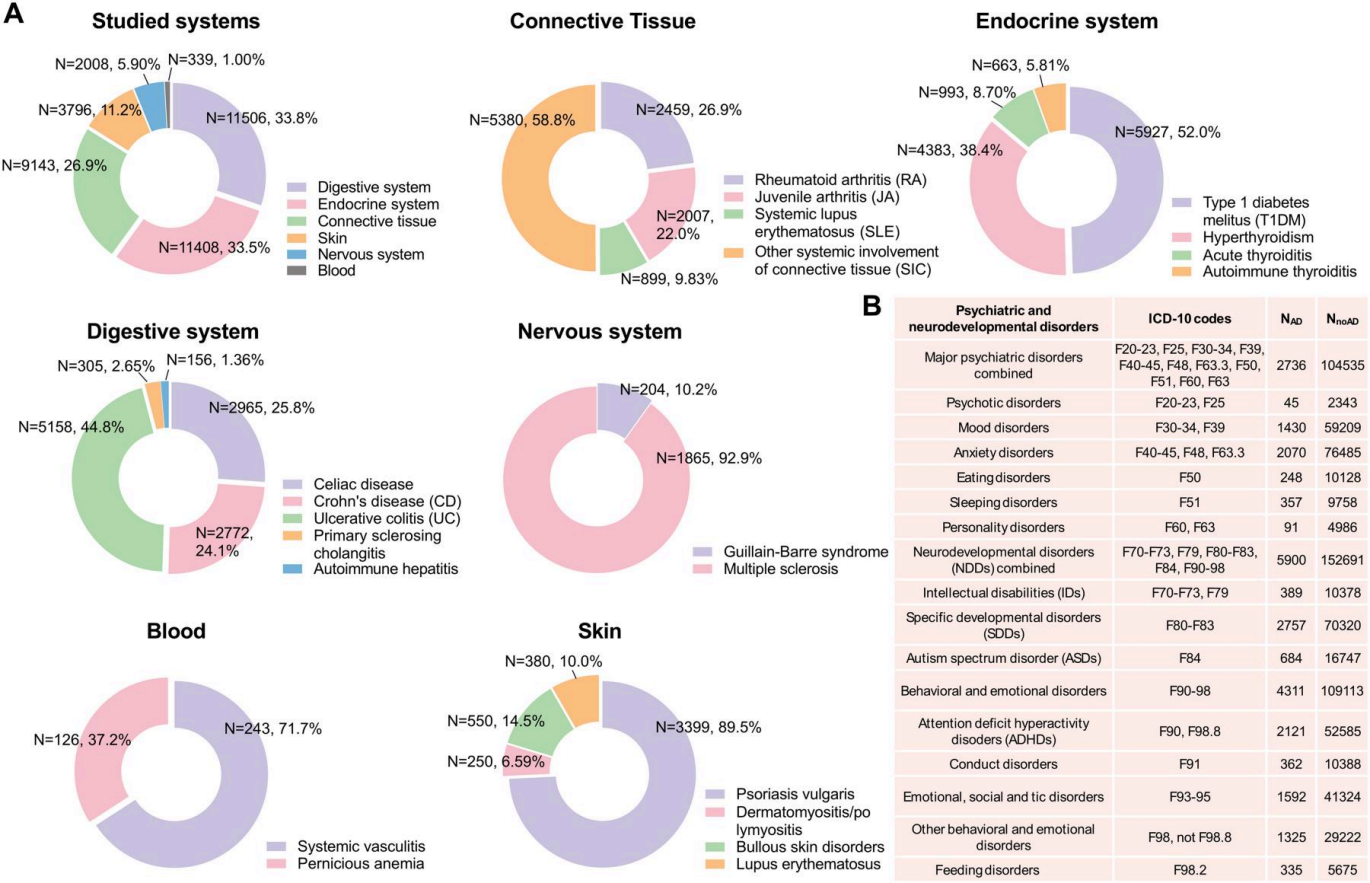
**Exposures and outcomes**. (**A**) Exposures: Maternal autoimmune and autoinflammatory disorders (ADs) grouped according to primary affected body system, including specific diagnoses and their respective number and proportion of offspring exposed prenatally. ICD-9 and ICD-10 codes for all ADs are listed in Supplementary Table S3. (**B**) Outcomes: Offspring psychiatric and neurodevelopmental disorder groups studied. N_AD_ refers to numbers of offspring with outcomes among those exposed to any maternal AD, while N_noAD_ refers to numbers of offspring with outcomes of those unexposed to any maternal AD. ICD-10 codes for the outcomes are listed also in Supplementary Table S4.

Primary exposure was maternal AD/AID diagnosis recorded before or during pregnancy, identified through MBR and HILMO, based on the assumption that AD/AIDs occurring before pregnancy remain during pregnancy. Diagnoses were grouped by the main affected body system (Fig. 1A, Supplementary Tables S2 and S3).

### Outcome

Offspring psychiatric and NDD diagnoses (ICD-10 F-diagnoses, F00–F98) were identified from HILMO (Fig. 1B, Supplementary Table S4). Due to the low age of the cohort, case numbers were small of organic mental disorders (F00–F09) and disorders due to psychoactive substance use (F10–F19). Therefore, these diagnoses were included only in the ‘any F-diagnosis’ outcome and not studied as specific outcomes.

### Covariates

Analyses were adjusted for birth year, sex, number of fetuses (1, 2, or ≥3), parity (0 or ≥1), maternal age at delivery (≤24, 25–34, or ≥35 years), marital status (married/cohabitating or unmarried/living alone), socioeconomic status (lower white-collar, blue-collar, other, upper white collar), maternal origin (born outside Finland; yes/no), maternal smoking during pregnancy (yes/no), maternal psychiatric history (any diagnosis) before pregnancy obtained from MBR and HILMO, and psychotropic medication (anatomic therapeutic chemical (ATC) codes: N05/N06) during pregnancy (yes/no) obtained from MBR or RRD. Missing data on smoking and socioeconomic status were coded as separate categories.

### Statistical analysis

Cox proportional hazards modeling was used to study the association between maternal AD/AIDs and offspring psychiatric disorders or NDDs. Proportional hazards assumptions were met. Exposures were grouped by the main affected body system for all AD/AIDs, with crude and adjusted hazard ratios (HRs) reported using 99% CIs (α = 0.01). Follow-up began at birth and ended December 2021 recording the first date of diagnosis. We had no information on emigration or death. To minimize risk of disease misclassification, two criteria were tested on the primary outcomes (major psychiatric disorders combined, NDDs combined): (i) a single registration of the outcome diagnosis, and (ii) the outcome diagnosis recorded at least twice. For blood system AD/AIDs, the HR point estimates for outcomes diagnosis were lower requiring only one registration, whereas for skin AD/AIDs, the HR point estimates for outcomes were higher requiring only one diagnosis registration. Since these differences between requiring one and two registrations were not statistically significant (Supplementary Table S5), analyses were conducted requiring only one record of outcome diagnosis. Associations for blood and skin AD/AIDs were tested also requiring two diagnosis registrations.

Significant associations were further examined in a *post hoc* analysis stratified by specific AD/AIDs (limited to those with ≥100 exposed births), using 95% CIs (α = 0.05). Outcomes with <5 cases were excluded.

To reduce misclassification risk of later-onset offspring diagnoses, minimum age thresholds were applied: ≥15 years for personality disorders, ≥10 years for psychotic, mood, and eating disorders, and ≥5 years for anxiety disorders.

Sensitivity analyses included (i) adjustment for AD-medication (ATC codes: A07E (intestinal anti-inflammatory agents), H02A (corticosteroids), L03A (immunostimulants), L04A (immunosuppressants other than corticosteroids), M01A (anti-inflammatory and antirheumatic agents, non-steroid), M01C (specific antirheumatic agents), N02B (analgesics and antipyretics) from RRD) dispensed 3 months before, during or 3 months after pregnancy for primary outcomes, (ii) stratification for markedly deviant proportions of cesarean section (CS) and/or PTB (before 37 weeks’ gestation) from MBR, and the influence of maternal obesity diagnosis prior to pregnancy (ICD-9 2781, 2780, 2788, 2599; ICD-10 E65–66 from HILMO and MBR).

All analyses were conducted using SAS version 9.4 (SAS Institute, Inc., Cary, NC, USA) between October 2023 and December 2025.

## Results

The incidence of births to mothers with any AD/AID (from now on called AD, Fig. 1A) increased over time, reaching a steady level around the year 2006 (Supplementary Table S1). Compared to unexposed pregnancies, those exposed to maternal AD differed significantly on most characteristics except sex and parity. Importantly, a higher proportion of mothers with AD had a psychiatric diagnosis before pregnancy (13.5% vs 6.7%) or used psychotropic medication during pregnancy (5.2% vs 2.9%). CS, PTB, and size for gestational age were largely similar between AD and non-AD groups, except for endocrine ADs, particularly type 1 diabetes (T1DM), where rates of CS, PTB, and LGA were higher (Supplementary Tables S2 and S6).

### Combined body system-related maternal ADs and offspring disorders

Exposure to any maternal AD was associated with higher risk of any psychiatric disorder or NDD (HR, 1.17 [99% CI, 1.13–1.20]) in offspring (Fig. 1B, Supplementary Table S7).

**Figure 2.**
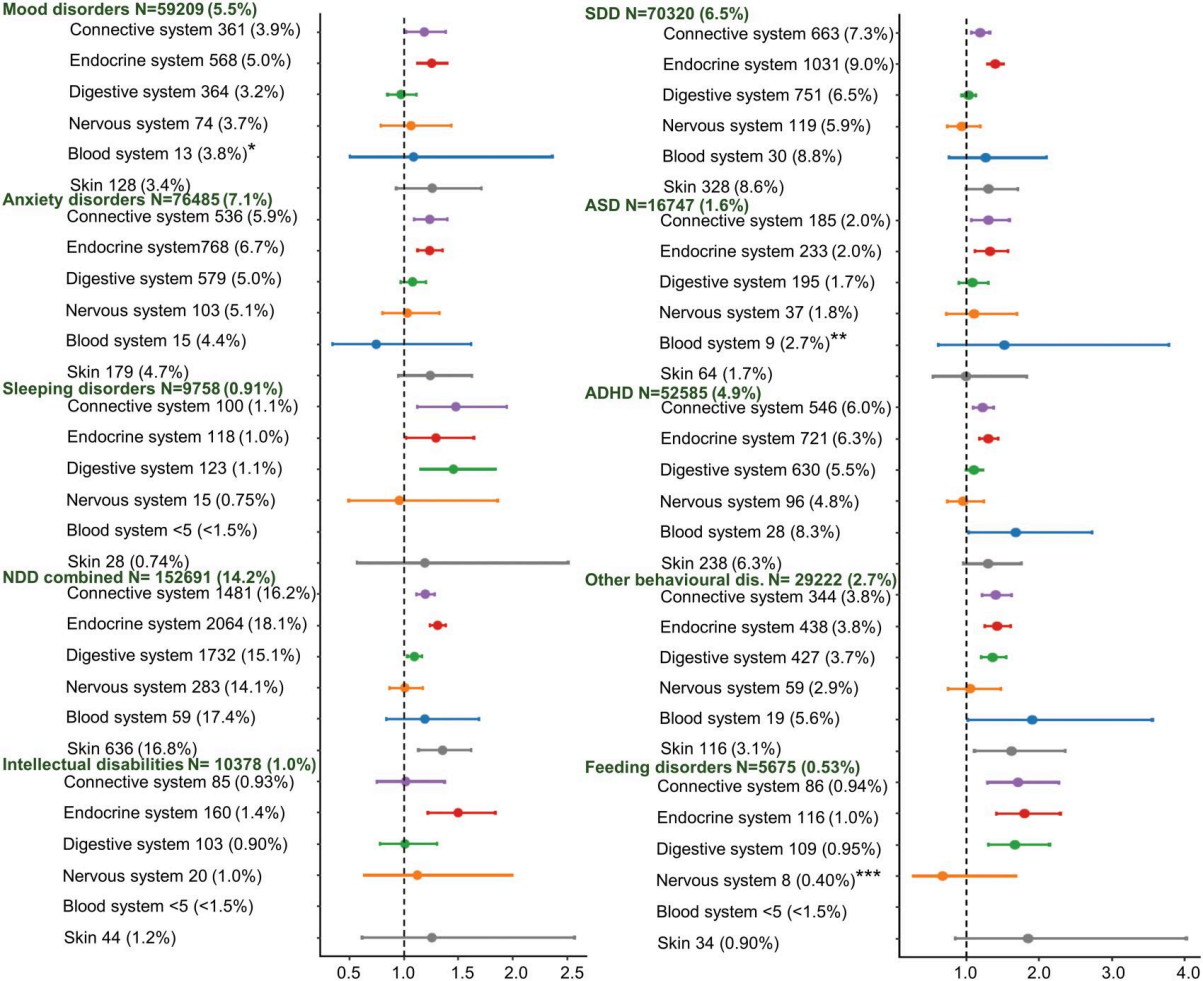
**Association analysis between prenatal exposure to maternal autoimmune and autoinflammatory disorder (AD) groups according to body system, and offspring psychiatric and neurodevelopmental disorders diagnosed from birth to December 2021 (hazard ratios with 99% CI)**. The exposure maternal disorder groups and outcome offspring disorder groups are defined in Fig. 1. ICD-9 and ICD-10 codes for all included ADs are listed in Supplementary Table S3. The number and (proportion) of offspring for each outcome among those not exposed to any maternal AD are given in green, and corresponding values for those exposed to an AD group are given in black. Analyses were performed using Cox proportional hazards modeling with adjustment for: Year of birth^*,**,***^, sex (boy/girl)^*,**^, multiple births (2 or ≥ 3)^***^, maternal age (≤ 24 or ≥35 years), parity (yes/no)^**,***^, marital status (married/cohabitating or unmarried/living alone), socioeconomic group (lower white-collar, blue-collar, other, upper white collar), migrant mother (yes/no)^***^, maternal smoking during pregnancy (yes/no) ^*^, maternal psychiatric disorder before pregnancy (any F diagnosis, in or out patient visits) ^*,**,***^, and psychotropic medication (N05/N06) during pregnancy (yes/no)^*,**,***^. Asterisks in graph indicate fewer covariates in the specific association test, including only those covariates with the corresponding aforementioned asterisks. Categories with N < 5 were not analyzed. NDD, neurodevelopmental disorders; SDD; specific developmental disorders ASD, autism spectrum disorder; ADHD, attention-deficit hyperactivity disorder.

Connective tissue-related ADs were associated with higher risks of major psychiatric disorders (HR, 1.18 [99% CI, 1.06–1.32]) and combined NDDs (HR, 1.20 [99% CI, 1.12–1.28]) in offspring, with larger effect sizes for mood disorders, anxiety disorders, sleeping disorder, specific developmental disorders (SDD), ASD, ADHD, and emotional, social, and tic disorders, other behavioral and emotional disorders (driven by incontinence and feeding disorders), and feeding disorders (HRs ranging from 1.19 to 1.71) (Fig. 2, Supplementary Table S7). Similarly, endocrine system-related ADs were associated with major psychiatric disorders (HR, 1.22 [99% CI, 1.13–1.33]) and combined NDDs (HR, 1.31 [99% CI, 1.24–1.39]), with larger effect sizes for mood disorders, anxiety disorders, sleeping disorder, intellectual disabilities (ID), SDD, ASD, ADHD, and emotional, social, and tic disorders, other behavioral and emotional disorders and feeding disorders (HRs ranging from 1.22 to 1.80).

Digestive system-related ADs were associated with a higher risk of combined NDDs (HR, 1.10 [99% CI, 1.03–1.17]), with larger effect sizes for other behavioral and emotional disorders (HR = 1.36), and feeding disorders (HR = 1.67), and higher risk was found for sleeping disorders (HR = 1.46). Blood system-related ADs showed higher risks for ADHD (HR = 1.68) and other behavioral and emotional disorders (HR = 1.91). No significant associations were found for nervous system-related ADs. However, skin-related ADs were associated with higher risk for combined NDDs (HR, 1.36 [99% CI, 1.13–1.62]), with higher risks for emotional, social, and tic disorders (HR = 1.46) and other behavioral and emotional disorders (HR = 1.62). Requiring two identical records of offspring outcome diagnosis, three new associations were detected for blood ADs: with combined NDDs 1.48 [1.05–2.11], SDD (HR = 1.95), and behavioral and emotional disorders (HR = 1.82) (Supplementary Table S7).

HRs being larger than expected from corresponding absolute risk differences could be explained by the earlier outcome onset in exposed than unexposed children (Supplementary Fig. S1).

### Specific maternal ADs and offspring disorders

Absolute numbers of exposure-outcome pairs are found in Supplementary Table S8.

#### Specific connective tissue ADs

**Figure 3.**
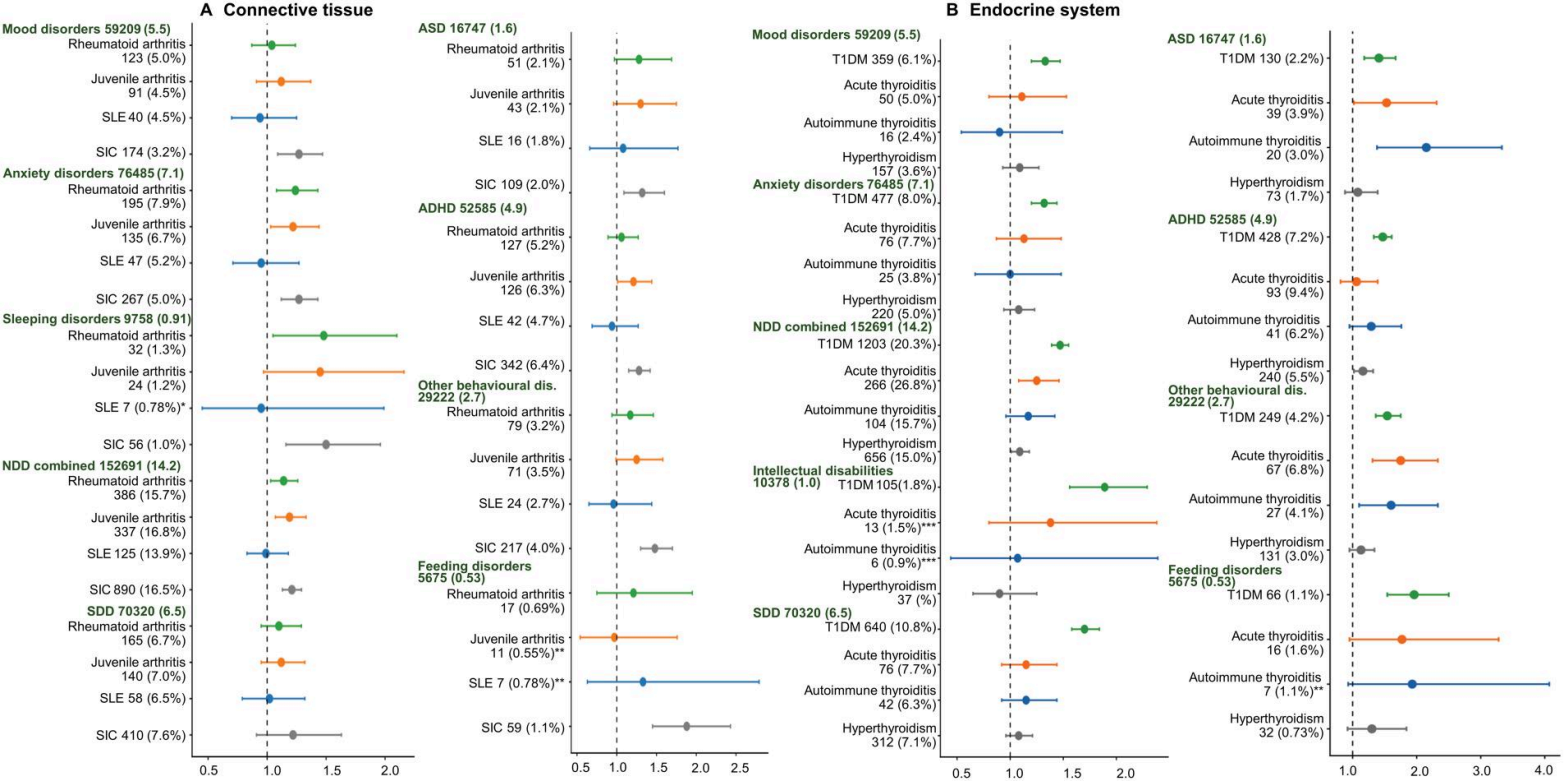
**Association analysis between prenatal exposure to maternal autoimmune and autoinflammatory disorder (AD) in connective tissue and the endocrine system, and offspring psychiatric and neurodevelopmental disorders (hazard ratios with 95% CI)**. (**A**) Connective tissue ADs include rheumatoid arthritis, juvenile arthritis, systemic lupus erythematosus (SLE), and other systemic involvement of connective tissue (SIC). (**B**) Endocrine system ADs include type 1 diabetes (T1DM), acute thyroiditis, autoimmune thyroiditis, and hyperthyroidism. Outcome offspring disorder groups, with diagnoses from birth to December 2021, are defined in Fig. 1B. The number and (proportion) of offspring for each outcome among those not exposed to any maternal AD are given in green, and corresponding values for those exposed to an AD group are given in black. Analyses for were performed using Cox proportional hazards modeling with adjustment for: Year of birth^*,**,***^, sex (boy/girl)^***^, multiple births (2 or ≥ 3)^*,**, ***^, maternal age (≤ 24 or ≥35 years), parity (0, ≥1), marital status (married/cohabitating or unmarried/living alone), socioeconomic group (lower white-collar, blue-collar, other, upper white collar)^^^, migrant mother (yes/no)^*,**, ***^, maternal smoking during pregnancy (yes/no)^*^, maternal psychiatric disorder before pregnancy (any F diagnosis, in or out patient visits) ^*,**^, and psychotropic medication (N05/N06) during pregnancy (yes/no)^*,**^. Asterisks in graph indicate fewer covariates in the specific association test, including only those covariates with the corresponding aforementioned asterisks. Categories with N < 5 were not analyzed. NDD, neurodevelopmental disorders; SDD; specific developmental disorders ASD, autism spectrum disorder; ADHD, attention-deficit hyperactivity disorder; SLE, systemic lupus erythematosus; SIC, other systemic involvement of connective tissue; T1DM, diabetes mellitus.

Maternal RA was associated with higher risks for major psychiatric disorders in offspring, with larger effect sizes for anxiety disorders (HR = 1.24) and sleeping disorders (HR = 1.48) (Fig. 3 and Supplementary Table S9), and also for combined NDDs, but not for individual NDDs. In contrast, maternal juvenile arthritis (JA) was associated with both anxiety disorders (HR = 1.22), behavioral–emotional disorders (HR = 1.26), and ADHD (HR = 1.21). Maternal SLE showed no association with offspring disorders, while systemic involvement of connective tissue (SIC) was associated with all offspring disorders studied except for SDD, with the largest pointwise effect sizes for sleeping disorders (HR = 1.50) and feeding disorders (HR = 1.88).

#### Specific endocrine ADs

Maternal T1DM was associated with major psychiatric disorders, with larger effect sizes for mood disorders (HR = 1.33) and anxiety disorders (HR = 1.32) in offspring. It was also associated with combined and all individual NDDs (HR ranging from 1.34 to 1.89) and feeding disorders (HR = 1.96) (Fig. 3 and Supplementary Table S10). Maternal acute thyroiditis was associated with higher risks of combined NDDs, with notable effects on ASD (HR = 1.53), behavioral–emotional disorders (HR = 1.31), and other behavioral and emotional disorders (HR = 1.75). Maternal autoimmune thyroiditis was associated with ASD (HR = 2.15) and other behavioral and emotional disorders (HR = 1.60). Maternal hyperthyroidism was associated with behavioral–emotional disorders (HR = 1.11) and ADHD (HR = 1.16).

#### Specific digestive system ADs

**Figure 4.**
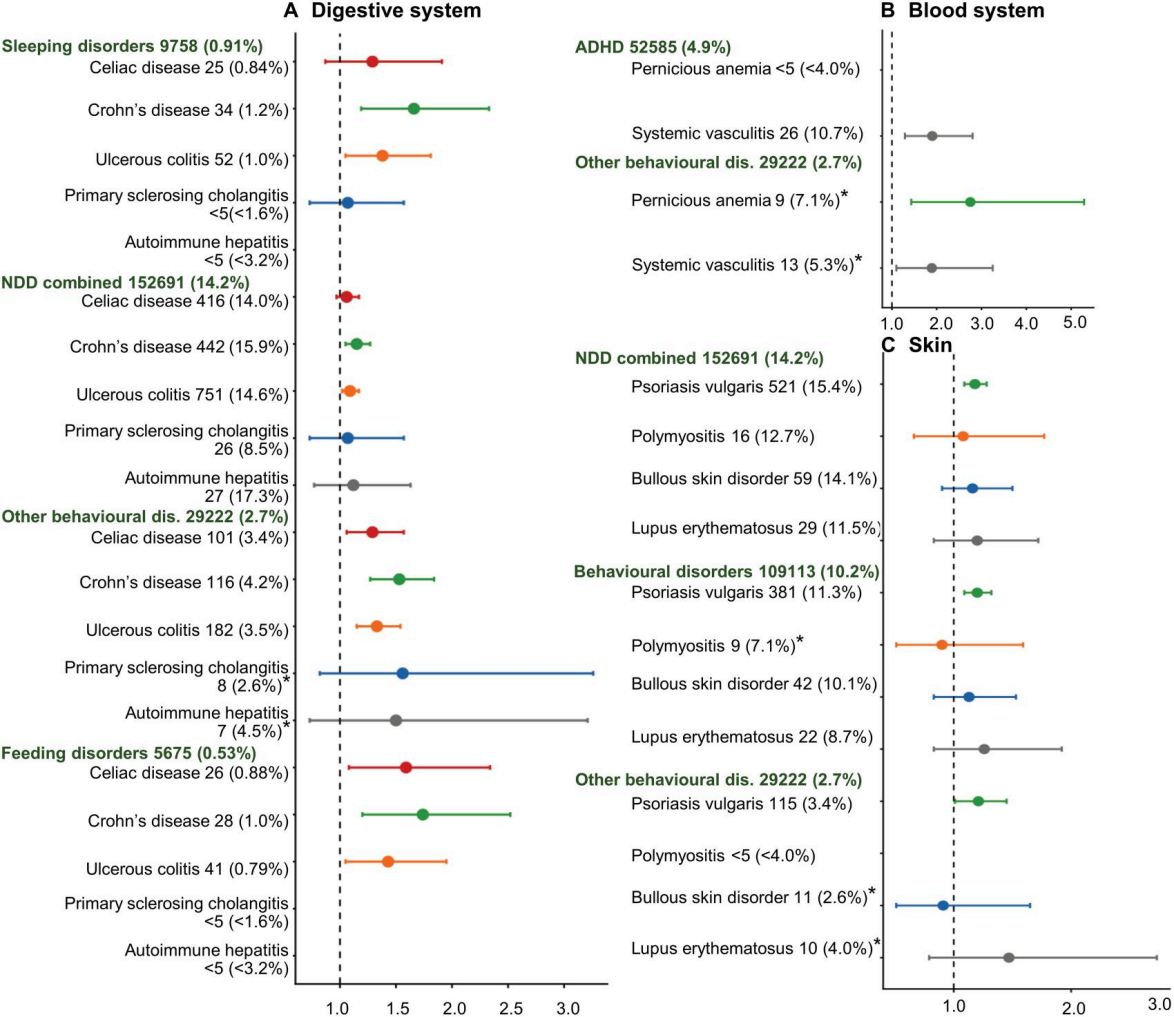
**Association analysis between prenatal exposure to maternal autoimmune and autoinflammatory disorder (AD) in the digestive system, blood system, and skin, and offspring psychiatric and neurodevelopmental disorders (hazard ratios with 95% CI)**. (**A**) Digestive system ADs include celiac disease, ulcerous colitis, Crohn’s disease, primary sclerosing cholangitis, and autoimmune hepatitis. (**B**) Blood system includes pernicious anemia and systemic vasculitis, and (**C**) skin includes Psoriasis vulgaris, polymyositis/dermatomyositis, bullous skin disorder, and lupus erythematosus. Outcome offspring disorder groups, with diagnoses from birth to December 2021, are defined in Fig. 1B. The number and (proportion) of offspring for each outcome among those not exposed to any maternal AD are given in green, and corresponding values for those exposed to an AD group are given in black. Analyses were performed using Cox proportional hazards modeling with adjustment for: Year of birth^*^, sex (boy/girl)^*^, multiple births (2 or ≥ 3)^*^, maternal age (≤ 24 or ≥35 years), first/only child (yes/no), marital status (married/cohabitating or unmarried/living alone), socioeconomic group (lower white-collar, blue-collar, other, upper white collar), migrant mother (yes/no), maternal smoking during pregnancy (yes/no), maternal psychiatric disorder before pregnancy (any F diagnosis, in or out patient visits)^*^, and psychotropic medication (N05/N06) during pregnancy (yes/no)^*^. Asterisk in graph indicates fewer covariates in the specific association test, including only those covariates with the corresponding aforementioned asterisk. Categories with N < 5 were not analyzed. NDD, neurodevelopmental disorders; ADHD, attention-deficit hyperactivity disorder.

Maternal celiac disease was associated only with other behavioral and emotional disorders in offspring (HR = 1.29) and its component feeding disorders (HR = 1.59) (Fig. 4 and Supplementary Table S11). In contrast, maternal Crohn’s disease (CD) was associated with psychiatric disorders, particularly sleeping disorders (HR 1.66, 95% CI, 1.19–2.33), and a higher risk for combined NDDs, with larger effect sizes for behavioral–emotional disorders (HR = 1.22), other behavioral and emotional disorders (HR = 1.53) and feeding disorders (HR = 1.74). Maternal ulcerative colitis was similarly associated with sleeping disorders (HR = 1.38) and combined NDDs, with larger effect sizes for behavioral–emotional disorders (HR = 1.14), other behavioral and emotional disorders (HR = 1.33) and feeding disorders (HR = 1.43). No association was found for maternal primary sclerosing cholangitis with offspring disorders, while autoimmune hepatitis was associated with behavioral–emotional disorders (HR 1.50).

#### Specific blood system ADs

Exposure to maternal pernicious anemia or systemic vasculitis was associated with higher risks of ADHD and other behavioral and emotional disorders (HRs ranging from 1.89 to 2.75) in offspring (Fig. 4 and Supplementary Table S12). Due to low sample size, analysis of outcomes requiring two diagnosis registrations was not performed.

#### Specific skin ADs

Maternal psoriasis vulgaris, but not polymyositis, bullous skin disorders or lupus erythematosus, was associated with higher risks for combined NDDs (HR = 1.18), particularly behavioral–emotional disorders (HR = 1.20) (Fig. 4 and Supplementary Table S13).

### Sensitivity analyses

**Figure 5.**
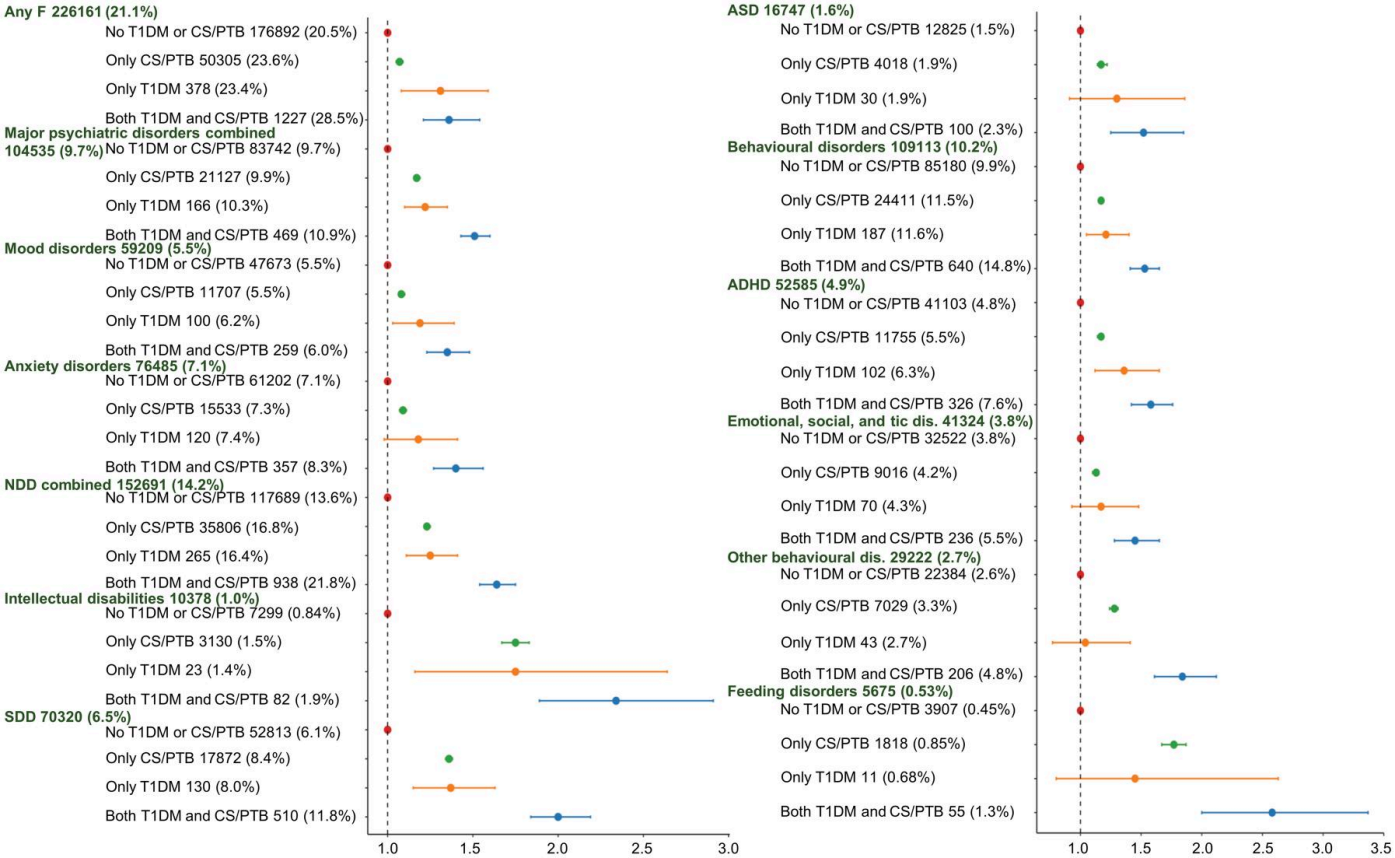
**Stratified analysis on presence of cesarean section and/or preterm birth (CS/PTB, yes/no) and maternal type 1 diabetes (T1DM (yes/no)) on the risk of offspring psychiatric and neurodevelopmental disorder (hazard ratios with 95% CI)**. Preterm birth was before gestational week 37. Births with no CS/PTB and no maternal T1DM was the reference group. Outcome offspring disorder groups, with diagnoses from birth to December 2021, are defined in Fig. 1B. The number and (proportion) of offspring for each outcome among those not exposed to any maternal AD are given in green, and corresponding values for those exposed to an AD group are given in black. Analyses were performed using Cox proportional hazards modeling with adjustment for: Year of birth, sex (boy/girl), multiple births (2 or ≥ 3), maternal age (≤ 24 or ≥35 years), first/only child (yes/no), marital status (married/cohabitating or unmarried/living alone), socioeconomic group (lower white-collar, blue-collar, other, upper white collar), migrant mother (yes/no), maternal smoking during pregnancy (yes/no), maternal psychiatric disorder before pregnancy (any F diagnosis, in or out patient visits), and psychotropic medication (N05/N06) during pregnancy (yes/no). CS, cesarean section; PTB, preterm birth; NDD, neurodevelopmental disorders; SDD; specific developmental disorders; ASD, autism spectrum disorder; ADHD, attention-deficit hyperactivity disorder; T1DM, diabetes mellitus.

Maternal AD-medication dispensation 3 months before (B3), in trimester (T1), T2, T3, or 3 months after pregnancy (A3), each had a small effect on offspring NDDs (99% CI of HR, 1.1–1.2), while A3 and B3 had a small effect also on major psychiatric disorders (Supplementary Tables S14, S15, and S16), but did not significantly influence the results in Figs 2–4. The CS/PTB-stratified analysis showed that maternal T1DM was associated with offspring psychiatric disorders and NDDs, even without CS/PTB. The risk was generally higher when both maternal T1DM and CS/PTB were present, being statistically significant for major psychiatric disorders combined, NDD combined, SDD, behavioral disorders, and other behavioral disorders (Fig. 5, Supplementary Table S17). Adjusting for maternal pre-pregnancy obesity did not alter the results (data not shown).

## Discussion

In this study, spanning over 16 million person-years, including 455 754 person-years with prenatal maternal AD exposure, we provide a comprehensive and detailed overview of associations between maternal ADs diagnosed prior to or during pregnancy and a broad range of psychiatric disorders and NDDs in offspring. To our knowledge, this is the first study to report an attempt to compare disease-specific effect sizes for nearly all known *in utero* AD exposures within a single cohort.

Most previous studies examining prenatal AD exposure and offspring mental health focused on a single exposure and/or outcome, mainly common ADs and ASD, and often lacked consideration of temporality (Vinet *et al.*, 2015; Wu *et al.*, 2015; Nielsen *et al.*, 2017; Mataix-Cols *et al.*, 2018; Rom *et al.*, 2018; Tsai *et al.*, 2018; Spann *et al.*, 2019; Chen *et al.*, 2021). Therefore, many associations between AD and psychiatric disorders identified in our study have not been previously reported. To limit multiple testing, we initially grouped maternal ADs according to the main body system involved. Associations with offspring NDDs and psychiatric diagnoses were primarily found for ADs involving connective tissues and endocrine systems, with modest effect sizes (HRs < 2), except for three notable exceptions, a more than 2-fold risks for (i) ASD in autoimmune thyroiditis and (ii) other behavioral or emotional disorders (F98) in pernicious anemia. We also observed 70–99% higher risk for ADHD and F98 in systemic vasculitis; for ID, SSD, and feeding disorders in T1DM; for F98 in acute thyroiditis; and for feeding disorders in CD and SIC (the contributors being polymyalgia rheumatica, Sjögren’s syndrome, hypermobility syndrome). Thus, ADs affecting primarily endocrine, blood, digestive, and connective systems were associated with NDDs and psychiatric disorders in offspring, with the child diagnoses with the highest effect sizes being NDDs and feeding disorders, which typically have onset in early childhood. Several additional associations were detected, however, with smaller effect sizes, but none in the AD group affecting primarily the nervous system.

### The specific findings in the context of previous knowledge

In our Finnish cohort, offspring exposed to any maternal AD had 17% higher risk of overall neurodevelopmental and psychiatric disorders (HR, 1.17 [99% CI, 1.13–1.20]), similar to findings from a Danish cohort (HR, 1.16 [95% CI, 1.13–1.19]) (He *et al.*, 2022). Our findings, grouping maternal ADs by body system and focusing on ‘any psychopathology’, generally support He *et al.*’s findings, who reported association for each AD group except for blood system (95% CI, 0.93–1.30). However, we detected no maternal nervous system AD association with offspring mental disorder (99% CI, 0.92–1.19), whereas He *et al.* (2022) reported a small effect size (95% CI, 1.07–1.29). Unlike He *et al.*, we also investigated associations between AD-system groups and specific childhood disorders, an analytical approach not comprehensively applied before, making direct comparisons with previous studies difficult. Nevertheless, the effect sizes we observed for specific common ADs and any offspring mental disorder were generally comparable to He *et al*., except for maternal SLE. They found higher risk with SLE (95% CI, 1.13–1.60), but we detected no significant association (95% CI, 0.86–1.16) (He *et al.*, 2022). This suggests that the overall *in utero* impact of maternal ADs, likely reflecting mild systemic immune activation, is modest on offspring psychiatric or neurodevelopmental health.

A 1.5- to 2-fold risk for ASD was observed after exposure to autoimmune thyroiditis (n_ASD among exposed_ = 20) or acute thyroiditis (n_ASD among exposed_ = 67). As maternal thyroid hormone is transported to fetus early in gestation (Adibi *et al.*, 2024), altered levels may disrupt fetal neurodevelopment (Zuñiga *et al.*, 2022; Adibi *et al.*, 2024). Presence of maternal thyroid autoantibody was previously reported to be associated with higher risk for offspring ASD (Brown *et al.*, 2015).

A nearly 3-fold risk for other behavioral or emotional disorders (F98) was found in offspring exposed to maternal pernicious anemia (n_F98 among exposed_ = 9), an association not previously reported. Pernicious anemia involves impaired vitamin B12 absorption due to autoimmune inflammation of the small intestine. Vitamin B12 is critical for neurodevelopment, especially during pregnancy and infancy, supporting processes such as neurotransmitter synthesis and myelination. Deficiency during pregnancy has been linked to delayed behavioral and cognitive development in humans (Cruz-Rodríguez *et al.*, 2023), and altered synaptic density in mice (Tat *et al.*, 2023). Systemic vasculitis was associated with a mildly higher risk for ADHD (n_ADHD among exposed_ = 26) and other behavioral or emotional disorders (n_F98 among exposed_ = 13). Pregnancies complicated by systemic vasculitis are considered high risk due to potential effects on blood vessels and placental function, which can impair nutrient and growth factor delivery to the fetus (Nguyen *et al.*, 2021). Notably, late first trimester and early second trimester, when maternal–fetal blood flow begins, are critical periods associated with NDD risk (Wen *et al.*, 2023).

Most associations with mental diagnoses in offspring were observed for maternal AD groups affecting (i) the connective tissue, with the most prevalent diagnoses being SIC, RA, and JA, and (ii) the endocrine system, with the most prevalent diagnosis being T1DM. Exposure to SIC was associated with all disorders but for SDD at modest effect size for feeding and sleeping disorders, while RA was associated with anxiety disorders, sleeping disorders, and NDD combined, at mild effect sizes. The effect of prenatal SIC exposure on offspring behavioral or psychiatric outcomes has not previously been reported. Maternal RA was not previously reported in relation to offspring anxiety or sleeping disorders. We found no association between maternal RA and offspring ASD (95% CI, 0.97–1.69), consistent with a systematic review and meta-analysis by Sun *et al*., including analysis restricted to RA diagnoses before or during pregnancy (Sun *et al.*, 2022). In contrast, exposure to maternal JA was associated with offspring anxiety (95% CI, 1.03–1.44), behavioral–emotional disorders (95% CI, 1.12–1.42), and ADHD (95% CI, 1.01–1.44). Although maternal JA has been associated with adverse birth outcomes (Ehrmann Feldman *et al.*, 2016), this was not observed in our cohort. We cannot exclude that some associations with maternal JA may reflect prodromal symptoms in the child, given the high co-occurrence of JA and anxiety (Delcoigne *et al.*, 2023). The higher risks for ID (95% CI, 1.56–2.29), ASD (95% CI, 1.18–1.67), and ADHD (95% CI, 1.33–1.61) in offspring exposed to maternal T1DM align with previous findings from a Swedish cohort (Chen *et al.*, 2021).

### Sensitivity analysis

Maternal ADs (e.g. systemic sclerosis, celiac disease, RA, JA, T1DM, SLE) have been associated with CS, preterm delivery, SGA, or LGA (Singh *et al.*, 2023). In our cohort, children exposed during pregnancy to maternal T1DM had higher rates of CS, PTB, and LGA. Exposure to CS and PTB, but not LGA, has previously been associated with psychiatric and NDDs (Zhang *et al.*, 2019; Kong *et al.*, 2025). Maternal T1DM was associated with offspring NDDs or psychiatric disorders even without CS and/or PTB, with similar risk estimates as CS and/or PTB alone. Exposure to both maternal T1DM and CS/PTB was associated with an even higher risk for major psychiatric disorders combined, NDD combined, SDD, behavioral disorders, and other behavioral disorders. Although preeclampsia partly mediates this risk (Bandoli *et al.*, 2020), only 2.4% of AD-exposed births had preeclampsia. Obesity is linked to low-grade inflammation and increased risk of offspring mental health issues, with genetic ties between body fat percentage and ADHD (Hübel *et al.*, 2019). Adjusting for maternal obesity did not affect our results (data not shown).

### Potential mechanisms

Although the etiologies of ADs and neurodevelopmental and psychiatric disorders are polygenic with some genetic correlations (e.g. ASD with T1DM, ADHD with RA and psoriasis) (Todd, 2010; Kumar *et al.*, 2019), associations between parental AD and offspring NDD disorders are strongest maternally (Chen *et al.*, 2021; He *et al.*, 2022; Hegvik *et al.*, 2022; Sun *et al.*, 2022). For example, maternal T1DM poses a higher risk for offspring NDD than paternal T1DM (Chen *et al.*, 2021). Although familial co-aggregation of ADHD and ADs suggests mild shared genetics, the strongest link remains maternal (Hegvik *et al.*, 2022). Interestingly, a negative genetic association between maternal T1DM and ADHD indicates that the higher ADHD risk observed with maternal T1DM exposure (95% CI, 1.33–1.61) cannot be fully explained by genetics alone (Tylee *et al.*, 2018), implying a role for the intrauterine environment.

The proposed mechanisms linking prenatal immune-mediated risk to mental disorders include the transfer of maternal autoantibodies across the placenta, which can target fetal brain proteins and disrupt neurodevelopment. Such autoantibodies have been associated with conditions like ASD in the offspring, suggesting a direct pathogenic role (Marks *et al.*, 2020; Ramirez-Celis *et al.*, 2022). Inflammatory mediators generated by the maternal immune response may also cross the placental barrier and interfere with fetal brain development (McLellan *et al.*, 2022). Further, an altered prenatal immune environment can lead to epigenetic modifications and changes in expression of genes critical for neurodevelopment (Bergdolt and Dunaevsky, 2019; McEwan *et al.*, 2023).

### Limitations

This study has several limitations. First, both maternal and paternal AD have previously been associated with offspring ADHD and ASD, although maternal AD had stably larger effect size than paternal AD in a large meta-analysis (Ellul *et al.*, 2022). We had no data on any paternal information or breastfeeding, and the number of exposure-discordant siblings was too small to adequately control for shared unmeasured familial confounding. Likewise, the possible influence of non-shared environmental factors, like school and peer relationships, could not be controlled for. Second, some ADs and maternal AD–offspring NDD pairs were rare, implying wide CIs of risk estimates, and difficulties to exclude false negative associations. Third, we cannot exclude rare prodromal AD-symptoms, and hence misclassification. Fourth, we cannot exclude differences in maternal health care-seeking behavior, and hence detection bias of offspring disorder. Fifth, our assumption that ADs recorded before pregnancy remained during pregnancy may not be valid for all cases, e.g. autoimmune thyroiditis, may resolve following thyroidectomy. Information on AD remission prior to pregnancy was not available. Sixth, grouping AD/AIDs by the main affected body system implies a risk for not detecting true associations of individual AD/AIDs.

Finally, the study is exploratory with multiple statistical tests (6 exposures and 17 partly inter-dependent outcomes), and the observational design and analytical approach preclude causal inference.

## Conclusion

The findings suggest mildly higher risks for several psychiatric and NDDs in offspring exposed to maternal AD. Notably, certain exposures were indicated to be associated with 2-fold risks, including ASD following exposure to autoimmune thyroiditis, and other behavioral or emotional disorders (F98) following exposure to pernicious anemia. The comprehensive inclusion of a broad spectrum of maternal ADs and offspring outcomes allowed comparative assessment of risk patterns across diagnostic groups. The findings may provide information for maternity care and family planning clinics potentially alleviating concerns among mothers with an AD/AID regarding offspring risk of neurodevelopmental and psychiatric disorders.

## Supplementary Material

hoag026_Supplementary_Data

## Data Availability

Data are available from the Finnish Social and Health Data Permit Authority upon approved permission application (www.findata.fi).
