# Supplementary material for: Associations of prenatal exposure to maternal autoimmune disorders with a wide spectrum of psychiatric and neurodevelopmental disorders in offspring—a nationwide cohort study
Source: Hum Reprod Open. 2026 Apr 26;2026(2):hoag026. doi: 10.1093/hropen/hoag026 (PMC13133672; doi:10.1093/hropen/hoag026)
Supplement: hoag026_Supplementary_Data [file hoag026_supplementary_data.pdf]

Supplementary material

Title: Associations of prenatal exposure to maternal autoimmune disorders with a wide spectrum of psychiatric and neurodevelopmental disorders in offspring - a nationwide cohort study

Authors: Elin Skott, Wenjie Cai, Miranda Stiernborg, Anna Fogdell-Hahn, MaiBritt Giacobini, Mika Gissler, Samson Nivins, Catharina Lavebratt

Supplementary Table S1 Characteristics of offspring and their mothers for all live births 1996-2014 in Finland. Maternal AD columns comprise of births to mothers with any studied autoimmune or autoinflammatory disorder (AD).
Supplementary Table S2 Characteristics of offspring and their mothers for all live births 1996-2014 in Finland grouped according to maternal autoimmune or autoinflammatory disorders (ADs) in specific body systems.
Supplementary Table S3 Classification of exposure; maternal autoimmune or autoinflammatory disorders (ADs) considered according to the ICD-9 and ICD-10 codes.
Supplementary Table S4 Classification of outcome; F diagnosis of offspring psychiatric and neurodevelopmental disorder considered according to ICD-10.
Supplementary Table S5 Comparison between having one or two outcome diagnosis records. Adjusted Hazard Ratios (HR) for offspring psychiatric and mild neurodevelopmental disorders until 2021 in relation to maternal autoimmune or autoinflammatory disorders (ADs) grouped by the main affected body system.
Supplementary Table S6 Mode of delivery, preterm birth and size for gestational age for all live births 1996-2014 in Finland in specific maternal endocrine system ADs.
Supplementary Table S7 Details to Figure 2. Adjusted Hazard Ratios (HR) for offspring psychiatric and mild neurodevelopmental disorders until 2021 in relation to maternal ADs.
Supplementary Table S8 Numbers of children with specific psychiatric and mild neurodevelopmental disorders until 2021 explored in Tables S9-S13 in relation to specific maternal ADs.
Supplementary Table S9 Details to Figure 3A: connective tissue ADs. Adjusted Hazard Ratios (HR) for offspring psychiatric and mild neurodevelopmental disorders until 2021 in relation to maternal connective tissue ADs.
Supplementary Table S10 Details to Figure 3B: endocrine system ADs. Adjusted Hazard Ratios (HR) for offspring psychiatric and mild neurodevelopmental disorders until 2021 in relation to maternal endocrine system ADs.
Supplementary Table S11 Details to Figure 4A: digestive system ADs. Adjusted Hazard Ratios (HR) for offspring psychiatric and mild neurodevelopmental disorders until 2021 in relation to maternal digestive system ADs.
Supplementary Table S12 Details to Figure 4B: blood system ADs. Adjusted Hazard Ratios (HR) for offspring psychiatric and mild neurodevelopmental disorders until 2021 in relation to maternal blood system ADs.
Supplementary Table S13 Details to Figure 4C: skin ADs. Adjusted Hazard Ratios (HR) for offspring psychiatric and mild neurodevelopmental disorders until 2021 in relation to maternal skin ADs.
Supplementary Table S14 Adjusted Hazard Ratios (HR) for the exposure maternal AD-medication on outcomes major psychiatric disorders and neurodevelopmental disorders (NDDs) in offspring.
Supplementary Table S15 Adjusted Hazard Ratios (HR) for outcome offspring psychiatric and mild neurodevelopmental disorders until 2021 in relation to the exposure maternal ADs, adjusted for maternal AD medication use during pregnancy.
Supplementary Table S16 Number of births exposed during pregnancy to maternal medication for AD.
Supplementary Table S17 Details to Figure 5. Adjusted HRs for offspring disorders until 2021 in relation to maternal T1DM stratified on cesarean section and/or preterm birth (CS/PTB, yes/no).

Supplementary Figure S1. Cumulative incidence of mood disorders, anxiety disorders and neurodevelopmental disorders (NDD) combined across age of the born children, stratified by exposure to maternal ADs.

Supplementary Table S1 Characteristics of offspring and their mothers for all live births 1996-2014 in Finland. Maternal AD columns comprise of births to mothers with any studied autoimmune or autoinflammatory disorder (AD).

Variable	No maternal AD		Maternal AD [a]		P-value	Variable	No maternal AD		Maternal AD [a]		P-value
	N=1 073 769	(%)	N=34 033	(%)			N=1 073 769	(%)	N=34 033	(%)	
**Offspring year of birth**					<0.001	**Maternal psychiatric disorder before pregnancy [c]**					<0.001
1996-2000	286 266	26.7	4018	11.8		Yes	71 448	6.7	4608	13.5	
2001-2005	275 157	25.6	7606	22.3		No	1 002 321	93.3	29 425	86.5	
2006-2010	287 641	26.8	11 321	33.3		**Maternal psychiatric disorder during pregnancy [c]**					<0.001
2011-2014	224 705	20.9	11 088	32.6		Yes	10 025	0.9	674	2.0	
**Offspring sex**					0.83	No	1 063 744	99.1	33 359	98.0	
Boy	549 058	51.1	17 451	51.3		**Maternal psychotropic medication before pregnancy [d]**					<0.001
Girl	524 711	48.9	16 582	48.7		Yes	165 008	16.4	8838	26.0	
**No. of offspring with any AD**	33 364	3.1	1898	5.6		No	908 761	84.6	25 195	74.0	
**Offspring age at AD diagnosis (years)**	9.0		7.2			**Maternal psychotropic medication during pregnancy [d]**					<0.001
Median (25th-75th percentile)	(4.8-14.0)		(4.1-11.4)			Yes	30 903	2.9	1778	5.2	
**Number of fetuses**					<0.001	No	1 042 866	97.1	32 255	94.8	
1	1 041 853	97.0	32 827	96.5		**Maternal preeclampsia [e]**					<0.001
2	31 285	2.9	1184	3.4		Yes	11 146	1.0	824	2.4	
≥ 3	631	0.1	22	0.1		No	1 062 623	99.0	33 209	97.6	
**Maternal age (years)**					<0.001	**Socioeconomic status**					<0.001
≤24	199 713	18.6	4892	14.4		Upper white-collar	179 208	16.7	5524	16.2	
25–34	675 755	62.9	21 514	63.2		Lower white-collar	383 337	35.7	12 706	37.3	
≥35	198 293	18.5	7627	22.4		Blue-collar	156 490	14.6	4342	12.8	
Missing	8	0.0	0	0.0		Other	187 883	17.5	5802	17.0	
**Parity**					0.015	Missing	166 851	15.5	5659	16.6	
0	629 246	58.6	19 682	57.8		**Maternal marital status**					<0.001
≥1	443 330	41.3	14 329	42.1		Married/cohabitating	959 816	89.4	30 575	89.9	
Missing	1193	0.1	22	0.1		Single	111 109	10.3	3414	10.0	
**Maternal country of birth**					<0.001	Missing	2844	0.3	44	0.1	
Finland	1 007 089	93.8	33 066	97.2		**In vitro fertilisation (IVF)**					
Other	66 680	6.2	967	2.8		Yes	25 616	2.39	1106	3.06	<0.001
**Maternal smoking**					<0.001	No	1 046 039	97.61	35 041	96.94	
No smoking	886 070	82.5	28 125	82.6		**Mode of delivery**					<0.001
Stopped in first trimester	39 790	3.7	1576	4.6		Vaginal	817 279	76.1	21 570	63.4	
Continued smoking during pregnancy	120 396	11.2	3 546	10.4		Instrumental	78 211	7.3	2667	7.8	
Missing	27 513	2.6	786	2.2		Planned CS	75 829	7.1	4696	13.8	
**Size for gestational age [b]**					<0.001	Other CS	101 034	9.4	5083	14.9	
Small (SGA)	34 171	3.2	1318	3.9		Missing data	1416	0.1	17	0.1	
Appropriate (AGA)	1 005 350	93.6	29 448	86.5		**Preterm birth [f]**					<0.001
Large (LGA)	29 628	2.8	3157	9.3		No	1 015 753	94.6	29 564	86.9	
Missing data	4620	0.4	110	0.3		Yes	58 016	5.4	4469	13.1	

Abbrevations: CS, Cesarean section; AD, Autoimmune or autoinflammatory disorder.

[a] Maternal ADs included in body systems see Table S3.
[b] Size for gestational age being a birth weight and/or length more than 2 standard deviations (SD) below the sex- and gestational age-specific reference mean (Sankilampi et al., 2013) according to the International Societies of Pediatric Endocrinology and the Growth Hormone Research Society (Clayton et al. 2007).
[c] Maternal psychiatric disorders: in-patient care due to mental health disorders before or during pregnancy according to ICD–8: 290–317 in 1969–1986, ICD–9: 290–319 in 1987–1995, and in- and out-patient care according to ICD–10: F00–F99 in-patient 1996–2014 and out-patient 1998-2014.
[d] Mothers' purchase of ATC N05 or N06 before/during pregnancy (antipsychotics, anxiolytics, hypnotics, sedatives, antidepressants, stimulants or nootropics).
[e] Maternal preeclampsia was identified by ICD-10 diagnosis code O11 or O14.
[f] In vitro fertilization ICD-10 Z31.2.
[g] Preterm birth, delivery before 37th week.

Supplementary Table S2 Characteristics of offspring and their mothers for all live births 1996-2014 in Finland grouped according to maternal autoimmune or autoinflammatory disorders (ADs) in specific body systems.

Variable	No maternal AD		Connective tissue AD		Digestive system AD		Blood AD		Nervous system AD		Skin AD		Endocrine system AD	
	N=1 073 769	(%)	N = 9143	(%)	N= 11 506	%	N= 339	%	N= 2008	(%)	N=3796	(%)	N=11 408	(%)
**Offspring year of birth**														
1996-2000	286 266	27%	892	10%	1144	10%	31	9%	203	10%	199	5%	1797	16%
2001-2005	275 157	26%	2042	22%	2473	21%	72	21%	433	22%	819	22%	2583	23%
2006-2010	287 641	27%	3056	33%	3900	34%	114	34%	731	36%	1401	37%	3610	32%
2011-2014	224 705	21%	3153	34%	3989	35%	122	36%	641	32%	1377	36%	3418	30%
**Offspring sex**														
Boy	549 058	51%	4655	51%	5970	52%	176	52%	1028	51%	1942	51%	5801	51%
Girl	524 711	49%	4488	49%	5536	48%	163	48%	980	49%	1854	49%	5607	49%
**Number of fetuses**														
1	1 041 853	97%	8801	96%	11 097	96%	335	99%	1945	97%	3 637	96%	10 997	96%
2-3	31 916	3%	342	4%	409	4%	4	1%	63	3%	159	4%	411	4%
**Maternal age (years)**														
≤24	199 713	19%	1323	14%	1620	14%	60	18%	174	9%	616	16%	1684	15%
25–34	675 755	63%	5654	62%	7422	65%	200	59%	1341	67%	2332	61%	7148	63%
≥35	198 293	19%	2166	24%	2464	21%	79	23%	493	25%	848	22%	2576	23%
Missing	0	0%	0	0%	0	0%	0	0%	0	0%	0	0%	0	0%
**Parity**														
0	629 246	59%	5123	56%	6690	58%	182	54%	1126	56%	2259	60%	6922	61%
≥1	443 330	41%	4016	44%	4805	42%	156	46%	881	44%	1535	40%	4482	39%
Missing	1193	0%	4	0.0	11	0%	1	0%	1	0%	0	0%	4	0%
**Maternal country of birth**														
Finland	1 007 089	94%	8947	98%	11 319	98%	329	97%	1977	98%	3676	97%	10 881	95%
Other	66 680	6%	196	2%	187	2%	10	3%	31	2%	120	3%	527	5%
**Maternal smoking**														
No smoking	886 070	83%	7634	83%	9635	84%	274	81%	1649	82%	2 762	73%	9283	81%
Stopped in first trimester	39 790	4%	431	5%	554	5%	21	6%	98	5%	272	7%	486	4%
Continued smoking during pregnancy	120 396	11%	882	10%	1062	9%	40	12%	210	10%	682	18%	1355	12%
Missing	27 513	3%	196	2%	255	2%	4	1%	51	3%	80	2%	284	2%
**Size for gestational age [b]**														
Small (SGA)	34 171	3%	458	5%	445	4%	23	7%	86	4%	158	4%	332	3%
Appropriate (AGA)	1 005 350	94%	8402	92%	10 664	93%	303	89%	1865	93%	3508	92%	8443	74%
Large (LGA)	29 628	3%	260	3%	360	3%	11	3%	52	3%	124	3%	2593	23%
Missing data	4620	0%	23	0%	37	0%	2	1%	5	0%	6	0%	40	0%
**Maternal psychiatric disorder before pregnancy [c]**														
Yes	71 448	7%	1272	14%	1548	13%	69	20%	298	15%	542	14%	1603	14%
No	1 002 321	94%	7871	86%	9958	87%	270	80%	1710	85%	3254	86%	9805	86%
**Maternal psychiatric disorder during pregnancy [c]**														
Yes	10 025	1%	201	2%	217	2%	10	3%	44	2%	78	2%	223	2%
No	1 063 744	99%	8942	98%	11 289	98%	329	97%	1964	98%	3718	98%	11 185	98%
**Maternal psychotropic medication before pregnancy [d]**														
Yes	165 008	15%	2 553	28%	3043	26%	117	35%	616	31%	1 110	29%	2733	24%
No	908 761	85%	6590	72%	8463	74%	222	65%	1392	69%	2686	71%	8675	76%
**Maternal psychotropic medication during pregnancy [d]**														
Yes	30 903	3%	514	6%	591	5%	19	6%	137	7%	222	6%	564	5%
No	1 042 866	97%	8629	94%	10915	95%	320	94%	1871	93%	3574	94%	10 844	95%
**Maternal preeclampsia (O11 or O14) [e]**														
Yes	11 146	1%	312	3%	207	2%	8	2%	29	1%	108	3%	822	7%
No	1 062 623	99%	8831	97%	11299	98%	331	98%	1979	99%	3 688	97%	10 586	93%
**Socioeconomic status**														
Upper white collar	179 208	17%	1574	17%	1898	16%	49	14%	374	19%	522	14%	1686	15%
Lower white collar	383 337	36%	3452	38%	4439	39%	119	35%	762	38%	1429	38%	4138	36%
Blue collar	156 490	15%	1057	12%	1442	13%	41	12%	195	10%	580	15%	1624	14%
Other	187 883	18%	1482	16%	1871	16%	61	18%	326	16%	604	16%	2099	18%
Missing data	166 851	16%	1578	17%	1856	16%	69	20%	351	17%	661	17%	1861	16%
**Maternal marital status**														
Married/cohabitating	959 816	90%	8181	89%	10 390	90%	296	87%	1844	92%	3367	89%	10 215	90%
Single	111 109	10%	952	10%	1107	10%	43	13%	163	8%	428	11%	1170	10%
Missing	2844	0%	10	0%	9	0%	0	0%	1	0%	1	0%	23	0%
**In vitro fertilisation (IVF)**														
Yes	25 616	2.4%	311	3.4%	374	3.2%	10	2.9%	64	3.2%	92	2.4%	319	2.8%
No	1 046 039	97.6%	8832	96.6%	11132	96.8%	329	97.1%	1944	96.8%	3704	97.6%	11 089	97.3%
**Mode of delivery**														
Vaginal	817 279	76%	6272	69%	8016	70%	240	71%	1396	70%	2701	71%	5735	50%
Instrumental	78 211	7%	771	8%	956	8%	28	8%	193	10%	311	8%	750	7%
Planned CS	75 829	7%	887	10%	1117	10%	31	9%	216	11%	300	8%	2589	23%
Other CS	101 034	9%	1209	13%	1412	12%	40	12%	203	10%	482	13%	2327	20%
Missing data	1416	0%	4	0%	5	0%	0	0%	0	0%	2	0%	7	0%
**Preterm birth[e]**														
Yes	58 016	5%	796	9%	1029	9%	28	8%	138	7%	287	8%	2652	23%
No	1 015 753	95%	8347	91%	10 477	91%	311	92%	1870	93%	3509	92%	8756	77%

Abbreviations: CS, Cesarean section; AD, Autoimmune disorder.

[a] Maternal ADs included in body systems see Table S3.

[b] Size for gestational age being a birth weight and/or length more than 2 standard deviations (SD) below the sex- and gestational age-specific reference mean (Sankilampi et al., 2013) according to the International Societies of Pediatric Endocrinology and the Growth Hormone Research Society (Clayton et al. 2007).
[c] Maternal psychiatric disorders: in-patient care due to mental health disorders before or during pregnancy according to ICD–8: 290–317 in 1969–1986, ICD–9: 290–319 in 1987–1995, and in- and out-patient care according to ICD–10: F00–F99 in-patient 1996–2014 and out-patient 1998-2014.
[d] Mothers' purchase of ATC N05 or N06 before/during pregnancy (antipsychotics, anxiolytics, hypnotics, sedatives, antidepressants, stimulants or nootropics).
[e] Maternal preeclampsia was identified by ICD-10 diagnosis code O11 or O14.
[f] In vitro fertilization ICD-10 Z31.2.
[g] Preterm birth, delivery before 37th week.

Supplementary Table S3 Classification of exposure; maternal autoimmune or autoinflammatory disorders (ADs) considered according to the ICD-9 and ICD-10 codes.

Body system	AD Diagnosis	ICD-10	ICD-9	Included in group
**Connective tissue**	Rheumatoid arthritis	M05, M06	714	yes
	Juvenile arthritis	M08, M45	7143, 7200A	yes
	Other systemic connective system disorders (SIC)	M35.0, M35.1, M35.2, M35.3, M35.4, M35.5, M35.8, M35.9	7102, 7108, 7112A, 725, 729, 2794	yes
	Systemic lupus erythematosus (SLE)	M32	7100A	yes
**Endocrine system**	Diabetes mellitus (T1DM)	E10		yes
	Hyperthyroidism	E05.0, E05.1, E05.2	2420A, 2421A, 2422A	yes
	Autoimmune thyroiditis	E06.3	2452A	yes
	Addison	E27.1-E27.2	2554A	no
	Acute thyroditis	E06.0-E06.2, E06.5, E06.9	2450A, 2451A, 2458X, 2459X	yes
**Digestive system**	Celiac disease	K90	5790A	yes
	Crohn’s disease (CD)	K50	5550, 5551, 5552, 5559	yes
	Ulcerative colitis (UC)	K51	556	yes
	Chronic atrophic gastritis	K29.4	535	no
	Primary biliary cirrhosis	K74.3	5716A	no
	Autoimmune hepatitis	K73.9, K75.4	5714	yes
	Primary sclerosing cholangitis	K83.0	5761X	yes
**Blood system**	Pernicious anemia	D51.0	281, 5351X	yes
	Systemic vasculitis	M30, M31	4460A	yes
**Nervous system**	Multiple sclerosis	G35	340, 4149X	yes
	Guillain-Barré syndrome	G61.0	3570A, 4100A	yes
**Skin**	Psoriasis vulgaris	L40, M070, M090	696	yes
	Polymyositis/ Dermatomyositis	M33	7103, 7104	yes
	Bullous skin disorders	L10, L12, L13	694	yes
	Lupus erythematosus	L93	6954A	yes

Diagnosis was excluded if number of births exposed was fewer than 100

Supplementary Table S4 Classification of outcome; F diagnosis of offspring psychiatric and neurodevelopmental disorder considered according to ICD-10.

Group and ICD-code	Area of difficulty example	Group and ICD-code	Area of difficulty example
**Psychotic disorders**		**Personality disorders**	
F20	Schizophrenia	F60	Borderline
F21	Schizotypal disorder	F63	Pyromania, kleptomania
F22	Delusional disorders	**Intellectual disabilities**	
F23	Brief psychotic disorder	F70	Mild intellectual disabilities
F25	Schizoaffective	F71	Moderate intellectual disabilities
**Mood disorders**		F72	Severe intellectual disabilities
F30	Manic episode	F73	Profound intellectual disabilities
F31	Bipolar disorder	F79	Unspecified intellectual disabilities
F32	Depressive	**Specific developmental disorders (SDD)**	
F33	Major depressive disorder	F80	Expressive language disorder
F34	Cyclothymic disorder	F81	Dyslexia
F39	Unspecified mood	F82	Motor function
**Anxiety disorders**		F83	Mixed developmental disorders
F40	Phobic anxiety	**Autism spectrum disorder**	
F41	Generalized anxiety	F84	ASD, Retts
F42	Obsessive-compulsive disorder	**Behavioral and emotional disorders**	
F43	Reaction to severe stress	F90, F98.8	ADHD, ADD
F44	Dissociative disorders	F91	Conduct disorder
F45	Hypochondriacal disorders	F92	Conduct and emotions
F48	Depersonalization - derealization syndrome	F93	Separation anxiety
F63.3	Trichotillomania	F94	Selective mutism
**Eating disorders**		F95	Tic disorder
F50	Eating disorders	F98, not F98.8	Feeding disorders, incontinence
**Sleeping disorders**		**Other behavioral and emotional disorders**	
F51	Insomnia, Sleep terrors	F98, not F98.8	Feeding disorders (F98.2), Incontinence (F98.0-F98.1)

Supplementary Table S5 Comparison between having one or two outcome diagnosis records. Adjusted Hazard Ratios (HR) for offspring psychiatric and mild neurodevelopmental disorders until 2021 in relation to maternal autoimmune or autoinflammatory disorders (ADs) grouped by the main affected body system.

		Any ICD-10 F-one diagnosis	Any ICD-10 F-two diagnoses	Major psychiatric disorders combined-one diagnosis	Major psychiatric disorders combined-two diagnoses	NDDs combined-one diagnosis	NDDs combined-two diagnoses
**No AD**	N (%)	226 161(21.06)		104 535(9.74)		152 691(14.22)	
**Connective tissue n=9 143**	N (%)	1974(21.59)	1215(13.29)	695(7.60)	429(4.69)	1481(16.20)	781(8.54)
	HR (99% CI)	1.19(1.12-1.26)	1.19(1.10-1.28)	1.18(1.06-1.32)	1.25(1.10-1.41)	1.20(1.12-1.28)	1.17(1.06-1.28)
**Endocrine system n=11 408**	N (%)	2745(24.06)	1681(14.74)	1033(9.06)	591(5.18)	2064(18.09)	1143(10.02)
	HR (99% CI)	1.27(1.21-1.34)	1.26(1.18-1.34)	1.22(1.13-1.33)	1.17(1.05-1.30)	1.31(1.24-1.39)	1.37(1.27-1.48)
**Digestive system n=11 506**	N (%)	2272(19.75)	1370(11.91)	757(6.58)	435(3.78)	1732(15.05)	903(7.85)
	HR (99% CI)	1.09(1.03-1.15)	1.08(1.00-1.15)	1.05(0.95-1.15)	1.04(0.92-1.17)	1.10(1.03-1.17)	1.07(0.99-1.17)
**Blood n=339**	N (%)	68(10.06)	53(15.63)	23(6.78)	15(4.42)	59(17.50)	45(13.27)
	HR (99% CI)	1.06(0.76-1.47)	1.35(0.95-1.92)	0.98(0.54-1.78)	1.21(0.62-2.36)	1.19(0.84-1.69)	1.71(1.17-2.52)
**Nervous system n=2 008**	N (%)	391(19.47)	236(11.75)	140(6.97)	83(4.13)	283(14.09)	155(7.72)
	HR (99% CI)	1.04(0.92-1.19)	1.02(0.87-1.21)	1.04(0.84-1.30)	1.05(0.79-1.40)	1.01(0.87-1.18)	1.04(0.85-1.28)
**Skin=3 796**	N (%)	798(21.02)	504(13.28)	241(6.35)	141(3.71)	636(16.75)	354(9.33)
	HR (99% CI)	1.27(1.09-1.49)	1.14(1.02-1.28)	1.25(0.99-1.58)	1.10(0.88-1.37)	1.36(1.13-1.62)	1.16(1.02-1.34)

Any ICD-10 F diagnosis includes F00-F98. Major psychiatric disorders includes F20–23, F25; F30–34, F39; F40–45; F50; F60, F63. Neurodevelopmental disorders (NDDs) includes F70–73, F79; F80–83; F84; F90–91; F93–95; F98.
Analyses were conducted using Cox proportional hazards models with 99% confidence intervals. The analysis was adjusted for year of birth, sex, multiple births, maternal age (≤24 or ≥35 years), parity, marital status, socioeconomic group (with upper white collar as the reference), maternal migrant status, maternal smoking during pregnancy, and maternal psychiatric disorder before pregnancy, and use of psychotropic medication during pregnancy. The minimum age for onset of diagnosis was 15 years for personality disorders, 10 years for psychotic disorders, mood disorders, and eating disorders, and 5 years for anxiety disorders.

Supplementary Table S6 Mode of delivery, preterm birth and size for gestational age for all live births 1996-2014 in Finland in specific maternal endocrine system ADs.

	No maternal AD		Type 1 diabetes		Acute thyroiditis		Autoimmune thyroiditis		Hyperthyroidism	
**Variable**	N=1 073 769	(%)	N = 5927	(%)	N= 991	%	N= 663	%	N= 4383	(%)
**Mode of delivery**										
Vaginal	817 279	76%	1795	30%	752	76%	487	73%	3134	72%
Instrumental	78 211	7%	363	6%	66	7%	55	8%	312	7%
Planned CS	75 829	7%	2086	35%	69	7%	41	6%	402	9%
Other CS	101 034	9%	1678	28%	104	10%	80	12%	534	12%
Missing data	1416	0%	5	0%	0	0%	0	0%	1	0%
**Preterm birth** [a]										
Yes	58 016	5%	2165	37%	71	7%	55	8%	395	9%
No	1 015 753	95%	3762	63%	920	93%	608	92%	3988	91%
**Size for gestational age** [b]										
Small (SGA)	34 171	3%	101	2%	37	4%	18	3%	185	4%
Appropriate (AGA)	1 005 350	94%	3418	58%	921	93%	609	92%	4004	91%
Large (LGA)	29 628	3%	2384	40%	31	3%	32	5%	179	4%
Missing data	4620	0%	24	0%	2	0%	4	1%	15	0%

Abbreviations: AD, Autoimmune or autoinflammatory disorder; CS, Cesarean section.

Endocrine disorders covered in the analysis comprise type 1 diabetes (ICD-10: E10); acute thyroiditis (ICD-10: E06.0, E06.1, E06.2, E06.5, E06.9; ICD-9: 2450, 2451, 2458, 2459, 2450A, 2451A, 2458X, 2459X); autoimmune thyroiditis (ICD-10: E06.3; ICD-9: 2452A); and hyperthyroidism (ICD-10: E05.0, E05.1, E05.2; ICD-9: 2420A, 2420, 2421, 2421A, 2422, 2422A).

[a] Preterm birth, delivery before 37th week.

[b] Size for gestational age being a birth weight and/or length more than 2 standard deviations (SD) below the sex- and gestational age specific reference mean (Sankilampi et al., 2013) according to the International Societies of Pediatric Endocrinology and the Growth Hormone Research Society (Clayton et al. 2007).

Supplementary Table S7 Details to Figure 2. Adjusted Hazard Ratios (HR) for offspring psychiatric and mild neurodevelopmental disorders until 2021 in relation to maternal ADs.

**Outcomes**	**All AD N=34 033**		**No AD N=1 073 769**		**Any AD**			**Any AD**			**Connective tissue N=9143**			
	N	%	N	%	Crude HR	99% CI		Adjusted HR	99% CI		N	Adjusted HR	99% CI	
Any disorder (Any F)	7 774	23	226 161	21	**1.28**	1.25	1.32	**1.17**	1.13	1.20	1974	**1.19**	1.12	1.26
**Major psychiatric disorders combined**	2736	8	104 535	10	**1.18**	1.12	1.24	**1.15**	1.09	1.21	695	**1.18**	1.06	1.32
Psychotic disorders*	45	0	2343	0	**1.09**	0.74	1.61	**1.16**	0.79	1.72	12	**1.10**	0.42	2.93
Mood disorders*	1430	4	59 209	6	**1.28**	1.19	1.37	**1.15**	1.07	1.23	361	**1.19**	1.02	1.38
Anxiety disorders*	2070	6	76 485	7	**1.34**	1.26	1.42	**1.17**	1.11	1.24	536	**1.24**	1.09	1.40
Eating disorder*	248	1	10 128	1	**1.20**	1.01	1.41	**1.05**	0.89	1.24	61	**0.96**	0.66	1.40
Sleeping disorder	357	1	9758	1	**1.33**	1.16	1.53	**1.30**	1.13	1.49	100	**1.48**	1.12	1.95
Personality disorder*	91	0	4986	0	**1.28**	0.97	1.67	**1.19**	0.90	1.56	23	**1.25**	0.64	2.44
**Neurodevelopmental disorders combined**	5900	17	152 691	14	**1.29**	1.25	1.34	**1.18**	1.14	1.22	1481	**1.20**	1.12	1.28
Intellectual disabilities	389	1	10 378	1	**1.15**	1.01	1.32	**1.19**	1.04	1.36	85	**1.02**	0.75	1.37
Specific developmental disorders	2757	8	70 320	7	**1.24**	1.18	1.31	**1.19**	1.13	1.25	663	**1.19**	1.07	1.32
Autism spectrum disorder	684	2	16 747	2	**1.35**	1.22	1.49	**1.21**	1.10	1.34	185	**1.31**	1.07	1.59
Behavioral and emotional disorders	4311	13	109 113	10	**1.36**	1.31	1.42	**1.20**	1.16	1.25	1120	**1.25**	1.16	1.36
ADHD	2121	6	52 585	5	**1.40**	1.32	1.48	**1.18**	1.12	1.25	546	**1.23**	1.09	1.38
Conduct disorders	362	1	10 388	1	**1.19**	1.03	1.36	**1.13**	0.99	1.30	87	**1.14**	0.86	1.52
Emotional, social and tic disorders	1592	5	41 324	4	**1.34**	1.26	1.43	**1.16**	1.09	1.24	419	**1.21**	1.06	1.38
Other behavioral and emotional disorders	1325	4	29 222	3	**1.43**	1.33	1.54	**1.34**	1.25	1.44	344	**1.41**	1.22	1.63
Feeding disorders	335	1	5675	1	**1.77**	1.53	2.04	**1.62**	1.40	1.87	86	**1.71**	1.29	2.28

Supplementary Table S7 continued.

Outcomes	Endocrine system N=11 408				Digestive system N=11 506				Blood system N=339				Nervous system N=2008				Skin N=3796			
	N	Adjusted HR	99% CI		N	Adjusted HR	99% CI		N	Adjusted HR	99% CI		N	Adjusted HR	99% CI		N	Adjusted HR	99% CI	
Any disorder (Any F)	2745	**1.27**	1.21	1.34	2272	**1.09**	1.03	1.15	68	**1.06**	0.76	1.47	391	**1.04**	0.92	1.19	798	**1.27**	1.09	1.49
**Major psychiatric disorders combined**	1033	**1.22**	1.13	1.33	757	**1.05**	0.95	1.15	23	**0.98**	0.54	1.78	140	**1.04**	0.84	1.30	241	**1.25**	0.99	1.58
Psychotic disorders^	18	**1.15**	0.62	2.11	11	**0.98**	0.45	2.13	<5	**NA**			<5	**NA**			<5	**NA**		
Mood disorders^	568	**1.26**	1.13	1.40	364	**0.97**	0.85	1.11	13	**1.09***	0.50	2.36	74	**1.07**	0.79	1.44	128	**1.26**	0.93	1.71
Anxiety disorders^	768	**1.24**	1.13	1.36	579	**1.08**	0.97	1.20	15	**0.75**	0.34	1.62	103	**1.03**	0.80	1.33	179	**1.24**	0.95	1.63
Eating disorder^	89	**1.08**	0.82	1.43	75	**1.05**	0.78	1.42	<5	**NA**			15	**1.10**	0.56	2.14	23	**1.49**	0.73	3.06
Sleeping disorder	118	**1.29**	1.02	1.64	123	**1.46**	1.15	1.84	<5	**NA**			15	**0.96**	0.49	1.86	28	**1.19**	0.57	2.51
Personality disorder^	30	**0.93**	0.58	1.49	25	**1.13**	0.67	1.90	<5	**NA**			6	**1.42**	0.50	4.08	10	**1.08**	0.34	3.42
**Neurodevelopmental disorders combined**	2064	**1.31**	1.24	1.39	1732	**1.10**	1.03	1.17	59	**1.19****	0.84	1.69	283	**1.01**	0.87	1.18	636	**1.36**	1.13	1.62
Intellectual disabilities	160	**1.50**	1.22	1.84	103	**1.01**	0.78	1.30	<5	**NA**			20	**1.12**	0.63	2.00	44	**1.26**	0.62	2.57
Specific developmental disorders	1031	**1.40**	1.29	1.52	751	**1.03**	0.94	1.13	30	**1.27^^**	0.76	2.10	119	**0.94**	0.74	1.19	328	**1.31**	1.00	1.71
Autism spectrum disorder	233	**1.33**	1.12	1.57	195	**1.09**	0.90	1.31	9	**1.52****	0.62	3.78	37	**1.11**	0.73	1.69	64	**1.00**	0.54	1.83
Behavioral and emotional disorders	1447	**1.29**	1.21	1.38	1297	**1.15**	1.07	1.23	45	**1.30^^**	0.88	1.93	216	**1.07**	0.89	1.27	456	**1.45**	1.18	1.77
ADHD	721	**1.30**	1.18	1.44	630	**1.11**	1.00	1.23	28	**1.68**	1.03	2.73	96	**0.96**	0.74	1.25	238	**1.30**	0.96	1.76
Conduct disorders	127	**1.25**	0.99	1.57	110	**1.11**	0.86	1.42	<5	**NA**			15	**0.86**	0.44	1.67	42	**1.14**	0.56	2.32
Emotional, social and tic disorders	540	**1.26**	1.12	1.41	458	**1.07**	0.95	1.21	22	**1.61**	0.92	2.82	85	**1.08**	0.81	1.42	171	**1.46**	1.06	2.01
Other behavioral and emotional disorders	438	**1.42**	1.26	1.61	427	**1.36**	1.20	1.55	19	**1.91**	1.02	3.56	59	**1.05**	0.75	1.47	116	**1.62**	1.11	2.36
Feeding disorders	116	**1.80**	1.41	2.29	109	**1.67**	1.30	2.14	<5	**NA**			8	**0.67*****	0.27	1.67	34	**1.85**	0.85	4.03

Abbreviations: ADHD, attention deficit hyperactivity disorder.

Analyses were conducted using Cox proportional hazards models with 99% confidence intervals. The analyses were adjusted for year of birth, sex, multiple births, maternal age (≤24 or ≥35 years), parity, marital status, socioeconomic group (with upper white collar as the reference), maternal migrant status, maternal smoking during pregnancy, and maternal psychiatric disorder before pregnancy, and use of psychotropic medication during pregnancy.

*Adjusted for year of birth, sex, maternal smoking, maternal psychiatric disorder before pregnancy, and use of psychotropic medication during pregnancy.
**Adjusted for year of birth, sex, parity, maternal psychiatric disorder before pregnancy, and use of psychotropic medication during pregnancy.
***Adjusted for year of birth, multiple births, maternal migrant status, maternal psychiatric disorder before pregnancy, and use of psychotropic medication during pregnancy. The other covariates were not adjusted for due to a small sample size (n<15) and no influence on the HR.
Events with fewer than five cases were excluded and are shown as NA in the table.

^The minimum age for onset of diagnosis was 15 years for personality disorders, 10 years for psychotic disorders, mood disorders, and eating disorders, and 5 years for anxiety disorders.
^^New statistically significant association when requiring two registrations of outcome diagnosis HR (99% CI): NDD: 1.48 (1.05-2.11); SDD: 1.95 (1.02-3.71); Behavioral and emotional disorders: 1.82 (1.09-3.05).

Maternal autoimmune disorders included exposures grouped by body system and corresponding ICD codes as follows: connective tissue system disorders including rheumatoid arthritis (ICD-10: M05, M06; ICD-9: 714), juvenile arthritis (ICD-10: M08, M45; ICD-9: 7143, 7200A), systemic lupus erythematosus (ICD-10: M32; ICD-9: 7100A), and other systemic involvement of connective tissue (ICD-10: M35.0, M35.1, M35.2, M35.3, M35.4, M35.5, M35.8, M35.9; ICD-9: 7102, 7108, 7112A, 725, 729, 2794). Endocrine disorders included type 1 diabetes (ICD-10: E10), acute thyroiditis (ICD-10: E06.0, E06.1, E06.2, E06.5, E06.9; ICD-9: 2450, 2451, 2458, 2459, 2450A, 2451A, 2458X, 2459X), autoimmune thyroiditis (ICD-10: E06.3; ICD-9: 2452A), hyperthyroidism (ICD-10: E05.0, E05.1, E05.2; ICD-9: 2420A, 2420, 2421, 2421A, 2422, 2422A), and Addison disease (ICD-10: E27.1–E27.2; ICD-9: 2554A). Digestive system disorders included celiac disease (ICD-10: K90; ICD-9: 5790A), Crohn’s disease (ICD-10: K50; ICD-9: 5550, 5551, 5552, 5559), ulcerative colitis (ICD-10: K51; ICD-9: 556), primary sclerosing cholangitis (ICD-10: K83.0; ICD-9: 5761X), autoimmune hepatitis (ICD-10: K73.9, K75.4; ICD-9: 5714), and primary biliary cirrhosis (ICD-10: K74.3; ICD-9: 5716A). Blood system disorders included pernicious anemia (ICD-10: D51; ICD-9: 281, 5351X) and systemic vasculitis (ICD-10: M30, M31; ICD-9: 4460A). Nervous system disorders included multiple sclerosis (ICD-10: G35, G61.0; ICD-9: 340, 4149X, 3570A, 4100A) and Guillain-Barré syndrome (ICD-10: G61.0; ICD-9: 3570A, 4100A). Skin system disorders included psoriasis vulgaris (ICD-10: L40, M07.0, M07.1, M07.2, M07.3; ICD-9: 696), dermatomyositis/polymyositis (ICD-10: M33; ICD-9: 7103, 7104), bullous skin disorders (ICD-10: L10, L12, L13; ICD-9: 694), and lupus erythematosus (ICD-10: L93; ICD-9: 6954A). Specific autoimmune disorders included in each body system group are listed in eTable 3.

Any offspring psychiatric and neurodevelopmental disorder outcomes (any F) included ICD-10 F00-F98. Major psychiatric disorders combined (F20–23, F25; F30–34, F39; F40–45, F63.3; F50; F60, F63), psychotic disorders (F20–23, F25), mood disorders (F30–34, F39), anxiety disorders (F40–45, F48, F63.3), eating disorders (F50), sleeping disorders (F51), personality disorders (F60.63), neurodevelopmental disorders combined (F70–73, F79; F80–83; F84; F90–91; F93–95; F98), intellectual disabilities (F70–73, F79), specific developmental disorders (F80–83), autism spectrum disorder (F84), behavioral and emotional disorders (F90–98), attention-deficit hyperactivity disorders (F90, F98.8), conduct disorders (F91), emotional disorders in childhood (F93–95), other behavioral and emotional disorders (F98, excluding F98.8), and Feeding disorders of infancy and childhood (F98.2).

Supplementary Table S8 Numbers of children with specific psychiatric and mild neurodevelopmental disorders until 2021 explored in Tables S9-S13 in relation to specific maternal ADs.

	**Connective tissue N=9143**				**Endocrine system N=11 408**				**Digestive system N=11 506**					**Blood N=339**		**Skin N=3976**			
**Outcomes**	Rheumatoid arthritis N=2459, NF=580	Juvenile arthritis N=2007, NF=463	Systemic lupus erythematosus N=899, NF=172	Other systemic involvement of connective tissue N=5380, NF=1126	Type 1 Diabetes N=5927, NF=1 605	Acute thyroiditis N= 993, NF=222	Autoimmune thyroiditis N=663, NF=123	Hyperthy roidism N=4383, NF=870	Celiac disease N=2965, NF=526	Crohn's disease N=2772, NF=568	Ulcerous colitis N=5158, NF=987	Primary sclerosing cholangitis N=305, NF=32	Autoimm une hepatitis N=156, NF=38	Pernicious anemia N=126, NF=25	Systemic vasculitis N=243, NF=50	Psoriasis vulgaris N=3399, NF= 491	Dermatomyo sitis/polymyo sitis N=250, NF=20	Bullous skin disorder N=550, NF=63	Lupus erythematosus N=380, NF=43
**Any disorder (Any F)**	580	463	172	1126	1605	222	123	870	526	568	987	32	38	25	50	751	22	89	52
**Major psychiatric disorders combined**	245	170	69	346	635	102	31	300	159	172	346	10	15	11	15	237	6	28	22
**Mood disorders***	123	91	40	174	359	50	16	157	88	75	156	6	9	6	10	135	<3	12	13
**Anxiety disorders***	195	135	47	267	477	76	25	220	109	135	275	6	11	7	9	176	5	21	14
**Sleeping disorder**	32	24	7	56	65	7	6	38	25	34	52	<3	<3	0	4	28	0	<3	4
**Neurodevel opmental disorders combined**	387	337	125	890	1203	266	104	656	416	442	751	26	27	21	44	521	16	59	29
**Intellectual disabilities**	30	19	6	47	105	13	6	37	19	32	46	<3	0	<3	<3	43	<3	3	<3
**Specific developme ntal disorders**	165	140	58	410	640	76	42	312	181	188	318	12	9	8	22	297	13	35	15
**Autism spectrum disorders**	51	43	16	109	130	39	20	73	43	49	95	5	0	3	6	59	<3	3	4
**Behavioral and emotional disorders**	276	262	92	678	827	200	77	463	316	334	555	20	26	17	34	381	9	42	22
**ADHD**	127	126	42	342	428	93	41	240	154	155	261	10	16	<3	26	224	6	24	9
**Emotional, social and tic disorders**	108	92	39	242	306	80	26	165	105	118	206	8	11	9	16	159	3	20	15
**Other behavioral and emotional disorders**	79	71	24	217	249	67	27	131	101	116	182	8	7	9	13	115	4	11	10
**Feeding disorders**	17	11	7	59	66	16	7	32	26	28	41	<3	<3	0	3	33	0	3	<3

Supplementary Table S9 Details to Figure 3A: connective tissue ADs. Adjusted Hazard Ratios (HR) for offspring psychiatric and mild neurodevelopmental disorders until 2021 in relation to maternal connective tissue ADs.

**Outcomes**	**Any connective tissue AD N=9143**		**No tissue AD N=1 073 769**		**Rheumatoid arthritis N=2459, NF=579**			**Juvenile arthritis N=2007, NF=463**			**Systemic lupus erythematosus (SLE) N=899, NF=172**			**Other systemic involvement of connective tissue (SIC) N=5380, NF=1126**		
	N	%	N	%	Adjusted HR	95% CI		Adjusted HR	95% CI		Adjusted HR	95% CI		Adjusted HR	95% CI	
Any disorder (Any F)	1974	22%	226 161	21%	**1.18**	1.09	1.29	**1.19**	1.09	1.31	**1.00**	0.86	1.16	**1.21**	1.14	1.28
**Major psychiatric disorders combined**	695	8%	104 535	10%	**1.14**	1.01	1.29	**1.13**	0.97	1.31	**1.05**	0.83	1.33	**1.22**	1.10	1.36
Mood disorders[¥]	361	4%	59 209	6%	**1.04**	0.87	1.24	**1.12**	0.91	1.37	**0.94**	0.70	1.25	**1.27**	1.09	1.47
Anxiety disorders[¥]	536	6%	76 485	7%	**1.24**	1.08	1.43	**1.22**	1.03	1.44	**0.95**	0.71	1.27	**1.27**	1.12	1.43
Sleeping disorder	100	1%	9758	1%	**1.48**	1.05	2.1	**1.45**	0.97	2.16	**0.95***	0.45	1.99	**1.50**	1.16	1.96
**Neurodevelopmental disorders combined**	1481	16%	152 691	14%	**1.14**	1.03	1.26	**1.19**	1.07	1.33	**0.99**	0.83	1.18	**1.21**	1.13	1.29
SDD	663	7%	70 320	7%	**1.10**	0.95	1.290	**1.12**	0.95	1.32	**1.02**	0.79	1.32	**1.19**	1.08	1.31
ASD	185	2%	16 747	2%	**1.28**	0.97	1.69	**1.30**	0.96	1.75	**1.08**	0.66	1.77	**1.32**	1.09	1.60
Behavioral and emotional disorders	1120	12%	109 013	10%	**1.12**	0.99	1.26	**1.26**	1.12	1.42	**1.01**	0.82	1.24	**1.29**	1.20	1.39
ADHD	546	6%	52 585	5%	**1.06**	0.89	1.27	**1.21**	1.01	1.44	**0.94**	0.69	1.27	**1.28**	1.15	1.42
Emotional, social and tic disorders	419	5%	41 324	4%	**1.13**	0.93	1.36	**1.15**	0.94	1.41	**1.10**	0.80	1.50	**1.21**	1.06	1.37
Other behavioral and emotional disorders	334	4%	29 222	3%	**1.17**	0.94	1.46	**1.25**	0.99	1.58	**0.96**	0.65	1.44	**1.48**	1.30	1.70
Feeding disorders	86	1%	5675	1%	**1.21**	0.75	1.95	**0.97****	0.54	1.76	**1.33****	0.63	2.79	**1.88**	1.45	2.43

Abbreviations: ADHD, Attention deficit hyperactivity disorder.

N (in bold) indicates the number of mothers with the exposure; NF (in bold) indicates the number of mothers with the exposure whose offspring had an F-diagnosis outcome.

The analysis included specific maternal Connective system autoimmune disorders (ADs) and only offspring psychiatric and neurodevelopmental outcomes that met the threshold for statistical significance at the 99% confidence interval (CI) level in the broader body system–level analysis. Cox proportional hazards models were used to estimate hazard ratios (HRs) and 95% CIs. Exposures with fewer than 100 cases were excluded from the analysis. Events with fewer than five cases were excluded and are shown as NA in the table.

Models were adjusted for year of birth, sex, multiple births, maternal age (≤24 or ≥35 years), parity, marital status, socioeconomic group (with upper white-collar as the reference), maternal migrant status, maternal smoking during pregnancy, and maternal psychiatric disorder before pregnancy, and use of psychotropic medication during pregnancy. Minimum age for onset of diagnosis was 10 years for mood disorders and 5 years for anxiety disorders.
*Adjusted for year of birth, multiple births, maternal migrant status, maternal smoking during pregnancy, maternal psychiatric disorder before pregnancy, and use of psychotropic medication during pregnancy.
**Adjusted for year of birth, multiple births, maternal migrant status, maternal psychiatric disorder before pregnancy, and use of psychotropic medication during pregnancy. The other covariates were not adjusted for due to a small sample size (n<15) and no influence on the HR.

Maternal Connective system autoimmune disorders (exposures) included Rheumatoid arthritis (ICD-10: M05, M06; ICD-9: 714), Juvenile arthritis (ICD-10: M08, M45; ICD-9: 7143, 7200A), Systemic lupus erythematosus (ICD-10: M32; ICD-9: 7100A), and Other systemic involvement of connective tissue (ICD-10: M35.0, M35.1, M35.2, M35.3, M35.4, M35.5, M35.8, M35.9; ICD-9: 7102, 7108, 7112A, 725, 729, 2794).

Any offspring psychiatric and neurodevelopmental disorder outcomes (any F) included ICD-10 F00-F98. Major psychiatric disorders combined (F20–23, F25; F30–34, F39; F40–45, F63.3; F50; F60, F63), Anxiety disorders (F40–45, F48, F63.3), Sleeping disorder (F51), Neurodevelopmental disorders combined (F70–73, F79; F80–83; F84; F90–91; F93–95; F98), Specific developmental disorders (F80–F83), Autism spectrum disorder (F84), Behavioral and emotional disorders (F90–F98), Attention-deficit hyperactivity disorders (F90, F98.8), Emotional disorders in childhood (F93–95), Other behavioral and emotional disorders (F98, excluding F98.8), and Feeding disorders of infancy and childhood (F98.2).
[¥]The minimum age for onset of diagnosis was 15 years for personality disorders, 10 years for psychotic disorders, mood disorders, and eating disorders, and 5 years for anxiety disorders.

Supplementary Table S10 Details to Figure 3B: endocrine system ADs. Adjusted Hazard Ratios (HR) for offspring psychiatric and mild neurodevelopmental disorders until 2021 in relation to maternal endocrine system ADs.

**Outcomes**	**Any endocrine AD N=11 408**		**No AD N=1 073 769**		**Type 1 Diabetes N=5927, NF=1605**			**Acute thyroiditis N=991, NF=222**			**Autoimmune thyroiditis N=663, NF=123**			**Hyperthyroidism N=4383, NF=870**		
	N	%	N	%	Adjusted HR	95% CI		Adjusted HR	95% CI		Adjusted HR	95% CI		Adjusted HR	95% CI	
Any disorder (Any F)	2745	24%	226 057	21%	**1.38**	1.32	1.45	**1.21**	1.06	1.38	**1.12**	0.94	1.34	**1.09**	1.02	1.17
**Major psychiatric disorders combined**	1033	9%	104 471	10%	**1.50**	1.48	1.53	**1.17**	0.94	1.47	**0.87**	0.61	1.25	**1.08**	0.97	1.21
Mood disorders[¥]	568	5%	59 171	6%	**1.33**	1.20	1.47	**1.11**	0.80	1.53	**0.90**	0.54	1.486	**1.09**	0.93	1.27
Anxiety disorders[¥]	768	7%	76 444	7%	**1.32**	1.20	1.44	**1.13**	0.87	1.48	**1.00**	0.67	1.48	**1.08**	0.94	1.23
**Neurodevelopmental disorders combined**	2064	18%	152 634	14%	**1.47**	1.39	1.55	**1.25**	1.08	1.46	**1.17**	0.96	1.42	**1.09**	1.01	1.18
Intellectual disabilities	160	1%	10 374	1%	**1.89**	1.56	2.29	**1.38***	0.82	2.35	**1.07***	0.48	2.39	**0.90**	0.65	1.25
Specific developmental disorders (SDD)	1031	9%	70 294	7%	**1.70**	1.58	1.84	**1.15**	0.92	1.44	**1.15**	0.92	1.44	**1.08**	0.96	1.21
Autism spectrum disorders (ASD)	233	2%	16 740	2%	**1.41**	1.18	1.67	**1.53**	1.02	2.31	**2.15**	1.38	3.33	**1.11**	0.88	1.39
Behavioral and emotional disorders	1447	13%	108 971	10%	**1.39**	1.30	1.49	**1.31**	1.10	1.55	**1.22**	0.97	1.53	**1.11**	1.01	1.21
ADHD	721	6%	52 565	5%	**1.47**	1.33	1.61	**1.06**	0.81	1.39	**1.29**	0.95	1.76	**1.16**	1.02	1.32
Emotional, social and tic disorders	540	5%	41 304	4%	**1.34**	1.20	1.50	**1.49**	1.15	1.94	**1.05**	0.70	1.58	**1.04**	0.89	1.21
Other behavioral and emotional disorders	438	4%	29 214	3%	**1.54**	1.36	1.75	**1.75**	1.31	2.33	**1.60**	1.10	2.33	**1.13**	0.95	1.34
Feeding disorders	116	1%	5675	1%	**1.96**	1.54	2.50	**1.77**	0.95	3.29	**1.93****	0.93	4.07	**1.30**	0.92	1.84

Abbreviations: ADHD, Attention-deficit hyperactivity disorder.
N (in bold) refers to the number of mothers with the exposure, and NF (in bold) refers to the number of mothers with the exposure whose offspring received an F-diagnosis outcome.
The analysis focused on specific maternal Endocrine system autoimmune disorders (ADs), and only outcomes that survived the 99% confidence interval threshold in the body system level analysis were included (Figure 2). A Cox proportional hazards model was used to estimate hazard ratios (HRs) with 95% confidence intervals (CI). Exposures with fewer than 100 cases were excluded (e.g., Addison's disease, N = 75).
The analyses were adjusted for year of birth, sex, multiple births, maternal age (≤24 or ≥35 years), first or only child status, single motherhood, socioeconomic group (with upper white-collar as the reference), migrant background, maternal smoking during pregnancy, and maternal psychiatric disorder before pregnancy, and use of psychotropic medication during pregnancy.

*Adjusted for year of birth, sex, multiple births, maternal migrant status and socioeconomic group. The other covariates were not adjusted for due to a small sample size (n<15) and no influence on the HR.
**Adjusted for year of birth, multiple births, maternal migrant status, maternal psychiatric disorder before pregnancy, and use of psychotropic medication during pregnancy. The other covariates were not adjusted for due to a small sample size (n<15) and no influence on the HR.

Exposures with fewer than 100 cases were excluded from the analysis. Events with fewer than five cases were excluded and are shown as NA in the table. Maternal Endocrine system autoimmune disorders (exposures) included: Type 1 diabetes (ICD-10: E10); acute thyroiditis (ICD-10: E06.0, E06.1, E06.2, E06.5, E06.9; ICD-9: 2450, 2451, 2458, 2459, 2450A, 2451A, 2458X, 2459X); autoimmune thyroiditis (ICD-10: E06.3; ICD-9: 2452A); and hyperthyroidism (ICD-10: E05.0, E05.1, E05.2; ICD-9: 2420A, 2420, 2421, 2421A, 2422, 2422A).

Any offspring psychiatric and neurodevelopmental disorder outcomes (any F) included ICD-10 F00-F98. Major psychiatric disorders combined (F20–23, F25; F30–34, F39; F40–45, F63.3; F50; F60, F63); Mood disorders (F30–34, F39); Anxiety disorders (F40–45, F48, F63.3); Neurodevelopmental disorders combined (F70–73, F79; F80–83; F84; F90–91; F93–95; F98); Intellectual disabilities (F70–73, F79); Specific developmental disorders (SDD, F80–83); Autism spectrum disorder (ASD, F84); Behavioral and emotional disorders (F90–F98); Attention-deficit hyperactivity disorders (F90, F98.8); Emotional disorders in childhood (F93–95); Other behavioral and emotional disorders (F98, excluding F98.8); and Feeding disorders of infancy and childhood (F98.2).
¥The minimum age for onset of diagnosis was 15 years for personality disorders, 10 years for psychotic disorders, mood disorders, and eating disorders, and 5 years for anxiety disorders.

Supplementary Table S11 Details to Figure 4A: digestive system ADs. Adjusted Hazard Ratios (HR) for offspring psychiatric and mild neurodevelopmental disorders until 2021 in relation to maternal digestive system ADs.

**Outcomes**	**Any digestive system AD N=11 506**		**No AD N=1 073 769**		**Celiac disease N=2965, NF=526**			**Crohn's disease N=2772, NF=568**			**Ulcerous colitis N=5158, NF=987**			**Primary sclerosing cholangitis N=305, NF=32**			**Autoimmune hepatitis N=156, NF=38**		
	N	%	N	%	Adjusted HR	95% CI		Adjusted HR	95% CI		Adjusted HR	95% CI		Adjusted HR	95% CI		Adjusted HR	95% CI	
Any disorder (Any F)	2272	25%	226 572	27%	**1.06**	0.97	1.15	**1.13**	1.04	1.23	**1.07**	1.01	1.14	**1.00**	0.70	1.41	**1.14**	0.83	1.57
Sleeping disorder	123	1%	9770	1%	**1.29**	0.87	1.91	**1.66**	1.19	2.33	**1.38**	1.05	1.81	**1.07**	0.73	1.57	**NA**		
**Neurodevelopmental disorders combined**	1732	18%	153 021	17%	**1.06**	0.97	1.17	**1.15**	1.05	1.27	**1.09**	1.02	1.17	**1.07**	0.73	1.57	**1.12**	0.77	1.63
Behavioral and emotional disorders	1297	13%	109 240	11%	**1.13**	1.00	1.26	**1.22**	1.09	1.35	**1.14**	1.05	1.24	**1.16**	0.75	1.80	**1.50**	1.02	2.21
Other behavioral and emotional disorders	427	4%	29 270	3%	**1.29**	1.06	1.57	**1.53**	1.27	1.84	**1.33**	1.15	1.54	**1.56***	0.78	3.13	**1.50***	0.71	3.14
Feeding disorders	109	1%	5690	0.5%	**1.59**	1.08	2.34	**1.74**	1.20	2.52	**1.43**	1.05	1.95	**NA**			**NA**		

N (in bold) refers to the number of mothers with the exposure, and NF (in bold) indicates the number of mothers with the exposure whose offspring received an F-diagnosis outcome. The analysis focused on specific maternal Digestive system autoimmune disorders (ADs) and included only outcomes that survived the 99% confidence interval threshold in the body system level analysis.

A Cox proportional hazards model was applied, with results presented as hazard ratios (HR) and 95% confidence intervals (CI). Exposures with fewer than 100 cases were excluded from analysis (e.g., primary biliary cirrhosis, N = 62). Outcomes with fewer than 5 events were excluded due to data protection policies and are presented as "NA" in the table.

The analyses were adjusted for year of birth, sex, multiple births, maternal age (≤24 or ≥35 years), parity, marital status, socioeconomic group (with upper white-collar as the reference), migrant background, maternal smoking during pregnancy, and maternal psychiatric disorder before pregnancy, and use of psychotropic medication during pregnancy.
*Adjusted for year of birth, sex, multiple births, maternal psychiatric disorder before pregnancy, and use of psychotropic medication during pregnancy. The other covariates were not adjusted for, due to a small sample size (n<15) and no influence on the HR.

Maternal Digestive system autoimmune disorders (exposures) included: celiac disease (ICD-10: K90; ICD-9: 5790A), Crohn's disease (ICD-10: K50; ICD-9: 5550, 555, 5552, 5559), ulcerative colitis (ICD-10: K51; ICD-9: 556), primary sclerosing cholangitis (ICD-10: K83.0; ICD-9: 5761X), and autoimmune hepatitis (ICD-10: K73.9, K75.4; ICD-9: 5714).

Any offspring psychiatric and neurodevelopmental disorder outcomes (any F) included ICD-10 F00-F98. Sleeping disorders (F51), Neurodevelopmental disorders combined (F70-73, F79; F80-83; F84; F90-91; F93-95; F98), Behavioral and emotional disorders (F90-F98), Other behavioral and emotional disorders (F98, excluding F98.8), and Feeding disorders of infancy and childhood (F98.2).

Supplementary Table S12 Details to Figure 4B: blood system ADs. Adjusted Hazard Ratios (HR) for offspring psychiatric and mild neurodevelopmental disorders until 2021 in relation to maternal blood system ADs.

**Outcomes**	**All blood AD N=339**		**No AD N=1 073 769**		**Pernicious anemia N=126, NF=25**			**Systemic vasculitis N=243, NF=50**		
	N	%	N	%	Adjusted HR	95% CI		Adjusted HR	95% CI	
ADHD	28	8%	52 717	5%	**NA**			**1.90**	1.29	2.80
Other behavioral and emotional disorders	19	6%	29 222	3%	**2.75***	1.43	5.29	**1.89***	1.10	3.25

Abbreviations: ADHD, Attention-Deficit/Hyperactivity Disorder.

N (in bold) indicates the number of mothers with the exposure; NF (in bold) represents the number of mothers with the exposure whose offspring received an F-diagnosis outcome.

The analysis focused on specific maternal Blood system autoimmune disorders (ADs) and included only outcomes that met the 99% confidence interval threshold in the body system level analysis. A Cox proportional hazards model was used, and results are presented with 95% confidence intervals (CI). Exposures with fewer than 100 cases were excluded. Outcomes with fewer than 5 events were omitted due to data protection policies and are indicated as "NA" in the table.

The models were adjusted for year of birth, sex, multiple births, maternal age (≤24 or ≥35 years), parity, marital status, socioeconomic group (with upper white-collar as the reference), maternal migration background, maternal smoking during pregnancy, and maternal psychiatric disorder before pregnancy, and use of psychotropic medication during pregnancy.
*Adjusted for year of birth, sex, multiple births, maternal psychiatric disorder before pregnancy, and use of psychotropic medication during pregnancy. The other covariates were not adjusted for, due to a small sample size (n<15) and no influence on the HR.

Maternal Blood system autoimmune disorders (exposures) included pernicious anemia (ICD-10: D51; ICD-9: 281, 5351X) and systemic vasculitis (ICD-10: M30, M31; ICD-9: 4460A). Offspring psychiatric and neurodevelopmental disorder outcomes included attention-deficit/hyperactivity disorder (ADHD; F90, with F98.8 included) and other behavioral and emotional disorders (F98, excluding F98.8).

Supplementary Table S13 Details to Figure 4C: skin ADs. Adjusted Hazard Ratios (HR) for offspring psychiatric and mild neurodevelopmental disorders until 2021 in relation to maternal skin ADs.

**Outcomes**	**Any skin AD N=3796**		**No AD N=1 073 769**		**Psoriasis vulgaris N=3373, NF=751**			**Dermatomyositis/polymyositis N=126, NF=22**			**Bullous skin disorder N=417, NF=89**			**Lupus erythematosus N=252, NF=52**		
	N	%	N	%	Adjusted HR	95% CI		Adjusted HR	95% CI		Adjusted HR	95% CI		Adjusted HR	95% CI	
Any disorder (Any F)	798	21%	226 161	21%	**1.15**	1.07	1.24	**0.93**	0.59	1.48	**1.16**	0.93	1.45	**1.10**	0.79	1.51
NDDs combined	636	17%	152 691	14%	**1.18**	1.09	1.28	**1.08**	0.66	1.77	**1.16**	0.90	1.50	**1.20**	0.83	1.72
Behavioral and emotional disorders	456	12%	109 013	10%	**1.20**	1.09	1.32	**0.90***	0.51	1.59	**1.13**	0.83	1.53	**1.26**	0.83	1.92
Emotional, social and tic disorders	171	5%	41 324	4%	**1.14**	0.97	1.33	**NA**			**1.17**	0.75	1.81	**1.58**	0.95	2.62
Other behavioral and emotional disorders	116	3%	29 222	3%	**1.21**	1.00	1.45	**NA**			**0.91***	0.51	1.65	**1.47***	0.79	2.73

Abbreviations: NDD, neurodevelopmental disorders. N (in bold): number with exposure. NF (in bold): number with both exposure and ICD-10 F-diagnosis outcome.

Analyses of specific maternal skin system autoimmune disorders include only outcomes that remained statistically significant at the 99% confidence interval (CI) level in the broader body system analysis. Cox proportional hazards models were used, and 95% confidence intervals are presented. Events with fewer than five cases were excluded due to data protection policies and are shown as NA in the table.

The analyses were adjusted for the following covariates: year of birth, sex, multiple births, maternal age (≤24 or ≥35 years), parity, marital status, socioeconomic group (upper white-collar as the reference), maternal migrant status, maternal smoking during pregnancy, and maternal psychiatric disorder before pregnancy, and use of psychotropic medication during pregnancy.
*Adjusted for year of birth, sex, multiple births, maternal psychiatric disorder before pregnancy, and use of psychotropic medication during pregnancy. The other covariates were not adjusted for, due to a small sample size (n<15) and no influence on the HR.

Maternal Skin system autoimmune disorders and ICD code (exposure) included: Psoriasis vulgaris (ICD-10: L40, M07.0, M07.1, M07.2, M07.3; ICD-9: 696); Dermatomyositis/polymyositis (ICD-10: M33; ICD-9: 7103, 7104); Bullous skin disorders (ICD-10: L10, L12, L13; ICD-9: 694) and Lupus erythematosus (ICD-10: L93; ICD-9: 6954A).
Any offspring psychiatric and neurodevelopmental disorder outcomes (any F) included ICD-10 F00-F98; Neurodevelopmental disorders combined (F70-73, F79; F80-83; F84; F90-91; F93-95; F98); Behavioral and emotional disorders (F90-F98); Emotional disorders in childhood (F93-95) and Other behavioral and emotional disorders (F98, F98.8 excluded).

Supplementary Table S14 Adjusted Hazard Ratios (HR) for the exposure maternal AD-medication on outcomes major psychiatric disorders and neurodevelopmental disorders (NDDs) in offspring.

**Offspring outcomes**	**3 months before pregnancy**			**Trimester 1**			**Trimester 2**			**Trimester 3**			**3 months after pregnancy**		
	Adjusted HR	99% CI		Adjusted HR	99% CI)		Adjusted HR	99% CI		Adjusted HR	99% CI		Adjusted HR	99% CI	
**Maternal connective tissue ADs: N=3357 on AD-medication/ 9143 with a connective tissue AD**															
Major psychiatric disorders combined	1.10	1.06	1.15	1.06	1.00	1.13	0.98	0.90	1.07	0.99	0.92	1.06	1.05	1.01	1.10
NDDs combined	1.14	1.10	1.17	1.15	1.11	1.20	1.19	1.13	1.25	1.16	1.11	1.21	1.14	1.11	1.17
**Maternal endocrine system ADs: N=2221/11 408**															
Major psychiatric disorders combined	1.11	1.06	1.15	1.07	1.01	1.14	0.99	0.91	1.08	1.00	0.93	1.07	1.06	1.01	1.11
NDDs combined	1.14	1.11	1.18	1.16	1.11	1.21	1.19	1.14	1.25	1.19	1.14	1.25	1.14	1.11	1.17
**Digestive system ADs: N=4436/11 506**															
Major psychiatric disorders combined	1.11	1.06	1.15	1.07	1.00	1.14	0.99	0.90	1.08	0.99	0.93	1.06	1.06	1.01	1.11
NDDs combined	1.14	1.11	1.17	1.15	1.11	1.20	1.19	1.13	1.25	1.16	1.12	1.21	1.14	1.11	1.17
**Blood system ADs: N=114/339**															
Major psychiatric disorders combined	1.11	1.06	1.16	1.07	1.01	1.14	0.99	0.91	1.08	1.00	0.93	1.07	1.06	1.01	1.11
NDDs combined	1.14	1.11	1.18	1.16	1.11	1.21	1.20	1.14	1.26	1.17	1.12	1.22	1.14	1.11	1.18
**Nervous system ADs: N=576/2008**															
Major psychiatric disorders combined	1.11	1.06	1.16	1.07	1.01	1.14	0.99	0.91	1.08	1.00	0.93	1.07	1.06	1.01	1.11
NDDs combined	1.14	1.11	1.18	1.16	1.11	1.21	1.20	1.14	1.26	1.17	1.12	1.22	1.14	1.11	1.18
**Skin ADs: N=897/3796**															
Major psychiatric disorders combined	1.11	1.06	1.16	1.07	1.01	1.14	0.99	0.91	1.08	1.00	0.93	1.07	1.06	1.01	1.11
NDDs combined	1.14	1.11	1.18	1.16	1.11	1.21	1.20	1.14	1.26	1.17	1.12	1.22	1.14	1.11	1.18

These models examined the association between maternal AD-medication use and offspring outcomes of major psychiatric disorders and neurodevelopmental disorders (NDDs). Effect sizes are presented as hazard ratios (HRs) with 99% confidence intervals (CIs) for each medication class. N refers to number on AD-medication/number with AD-diagnosis.

Maternal autoimmune and autoinflammatory disease (AD)-related medications identified using Anatomical Therapeutic Chemical (ATC) classification codes A07E (intestinal anti-inflammatory agents), H02A (corticosteroids), L03A (immunostimulants), L04A (immunosuppressants other than corticosteroids), M01A (non-steroidal anti-inflammatory and antirheumatic agents), M01C (specific antirheumatic agents), and N02B (analgesics and antipyretics). Model covariates were those included in the models presented in Figure 2 and TableS7.

Any ICD-10 F diagnosis includes F00-F98. Major psychiatric disorders includes F20–23, F25; F30–34, F39; F40–45; F50; F60, F63. Neurodevelopmental disorders (NDDs) includes F70–73, F79; F80–83; F84; F90–91; F93–95; F98.

Supplementary Table S15 Adjusted Hazard Ratios (HR) for outcome offspring psychiatric and mild neurodevelopmental disorders until 2021 in relation to the exposure maternal ADs, adjusted for maternal AD medication use during pregnancy.

		**Any disorder (Any F)**			**Major psychiatric disorders**			**Any neurodevelopmental disorder**		
		Adjusted HR	99% CI		Adjusted HR	99% CI		Adjusted HR	99% CI	
**Any AD**	Model 1	1.17	1.13	1.20	1.18	1.12	1.24	1.29	1.25	1.34
	Model 2(B3)	1.15	1.12	1.19	1.14	1.08	1.20	1.17	1.13	1.21
	Model 2(T1)	1.15	1.12	1.19	1.14	1.09	1.20	1.17	1.13	1.21
	Model 2(T2)	1.15	1.12	1.19	1.15	1.10	1.21	1.16	1.12	1.20
**Connective tissue n=9143**	Model 1	1.19	1.12	1.26	1.18	1.06	1.32	1.20	1.12	1.28
	Model 2(B3)	1.16	1.09	1.23	1.16	1.05	1.28	1.15	1.08	1.23
	Model 2(T1)	1.16	1.09	1.23	1.17	1.06	1.29	1.15	1.08	1.23
	Model 2(T2)	1.16	1.09	1.23	1.18	1.07	1.30	1.15	1.08	1.23
**Endocrine system n=11 408**	Model 1	1.27	1.21	1.34	1.22	1.13	1.33	1.31	1.24	1.39
	Model 2(B3)	1.26	1.20	1.33	1.22	1.13	1.32	1.30	1.23	1.38
	Model 2(T1)	1.26	1.20	1.33	1.22	1.13	1.33	1.30	1.23	1.38
	Model 2(T2)	1.27	1.20	1.33	1.22	1.13	1.33	1.30	1.23	1.38
**Digestive system n=11 506**	Model 1	1.09	1.03	1.15	1.05	0.95	1.15	1.10	1.03	1.17
	Model 2(B3)	1.06	1.01	1.12	1.03	0.94	1.13	1.07	1.00	1.14
	Model 2(T1)	1.06	1.00	1.12	1.03	0.94	1.14	1.06	1.00	1.13
	Model 2(T2)	1.06	1.00	1.12	1.05	0.95	1.15	1.06	0.99	1.13
**Blood system n=339**	Model 1	1.06	0.76	1.47	0.98	0.54	1.78	1.19	0.84	1.69
	Model 2(B3)	1.05	0.77	1.44	1.06	0.62	1.81	1.19	0.85	1.67
	Model 2(T1)	1.05	0.77	1.43	1.06	0.62	1.82	1.19	0.85	1.66
	Model 2(T2)	1.05	0.77	1.44	1.07	0.63	1.83	1.19	0.85	1.66
**Nervous system n=2008**	Model 1	1.04	0.92	1.19	1.04	0.84	1.30	1.01	0.87	1.18
	Model 2(B3)	1.02	0.90	1.16	1.03	0.83	1.28	0.99	0.85	1.15
	Model 2(T1)	1.03	0.90	1.17	1.04	0.83	1.29	0.99	0.85	1.16
	Model 2(T2)	1.03	0.91	1.18	1.04	0.84	1.29	1.00	0.86	1.16
**Skin n=3796**	Model 1	1.27	1.09	1.49	1.25	0.99	1.58	1.36	1.13	1.62
	Model 2(B3)	1.11	1.01	1.22	1.06	0.90	1.26	1.13	1.02	1.25
	Model 2(T1)	1.11	1.01	1.22	1.07	0.90	1.26	1.13	1.02	1.25
	Model 2(T2)	1.11	1.01	1.22	1.07	0.90	1.26	1.13	1.02	1.25

Analyses were conducted using Cox proportional hazards models with 99% confidence intervals.
Model 1 covariates were those included in Figure 2 and TableS7: year of birth, sex, multiple births, maternal age (≤24 or ≥35 years), parity, marital status, socioeconomic group (with upper white collar as the reference), maternal migrant status, maternal smoking during pregnancy, and maternal psychiatric disorder before pregnancy, and use of psychotropic medication during pregnancy.
Model 2(B3) included the Model 1 covariates plus maternal AD-medication dispensed during the 3 months before pregnancy (B3).
Model 2(T1) included the Model 1 covariates plus maternal AD-medication dispensed during trimester 1 (T1).
Model 2(B3) included the Model 1 covariates plus maternal AD-medication dispensed during trimester 2 (T2).

Any ICD-10 F diagnosis includes F00-F98. Major psychiatric disorders includes F20–23, F25; F30–34, F39; F40–45; F50; F60, F63. Neurodevelopmental disorders (NDDs) includes F70–73, F79; F80–83; F84; F90–91; F93–95; F98.

Supplementary Table S16 Number of births exposed during pregnancy to maternal medication for AD.

Exposure period	A07E	H02A	L03A	L04A	M01A	M01C	N02B	Any med.
**Maternal connective tissue ADs N=9143**								
B3	727	495	3	229	887	69	169	1886
Trimester 1	576	458	<3	129	359	22	209	1331
Trimester 2	517	549	0	80	159	0	290	1251
Trimester 3	645	751	<3	95	217	<3	470	1619
A3	507	645	<3	169	906	33	515	1854
Anytime B3-A3	1123	1291	5	335	1818	97	1074	3347
**Maternal endocrine system ADs N=11 408**								
B3	54	102	5	18	541	<3	101	741
Trimester 1	50	78	<3	18	250	0	113	465
Trimester 2	46	82	0	13	54	0	153	328
Trimester 3	57	117	0	16	82	0	265	493
A3	40	74	0	16	938	<3	659	1278
Anytime B3-A3	85	240	5	28	1576	<3	1005	2221
**Digestive system ADs N=11 506**								
B3	1713	360	6	412	530	0	179	2 500
Trimester 1	1700	303	<3	374	196	0	182	2 216
Trimester 2	1895	420	<3	396	70	0	195	2 334
Trimester 3	2303	673	<3	461	98	0	346	2 920
A3	1718	421	3	376	746	0	547	2 768
Anytime B3-A3	2707	1091	7	625	1331	0	1026	4 436
**Blood system ADs N=339**								
B3	4	24	0	13	19	0	7	51
Trimester 1	4	14		8	14	0	9	40
Trimester 2	<3	21	0	9	4	0	10	39
Trimester 3	4	25	0	9	6	0	16	53
A3	3	21	<3	10	34	0	24	64
Anytime B3-A3	5	48	<3	17	55	0	42	114
**Nervous system Ads**								
B3	8	20	199	4	158	0	29	340
Trimester 1	7	15	87	5	54	0	24	172
Trimester 2	8	7	13	3	18	0	33	75
Trimester 3	11	13	20	3	23	0	60	116
A3	9	29	90	4	146	0	92	258
Anytime B3-A3	14	62	271	7	319	0	174	576
**Skin ADs N=3796**								
B3	100	61	4	54	238	<3	58	414
Trimester 1	79	41	<3	29	99	<3	51	258
Trimester 2	72	51	0	17	21	0	91	212
Trimester 3	92	81	<3	20	33	0	150	299
A3	75	69	<3	37	299	3	182	477
Anytime B3-A3	141	158	4	72	563	4	363	897

Maternal AD-medication was identified using Anatomical Therapeutic Chemical (ATC) codes from the RRD: A07E (intestinal anti-inflammatory agents), H02A (corticosteroids), L03A (immunostimulants), L04A (immunosuppressants other than corticosteroids), M01A (non-steroidal anti-inflammatory and antirheumatic agents), M01C (specific antirheumatic agents), and N02B (analgesics and antipyretics).

Supplementary Table S17 Details to Figure 5. Adjusted HRs for offspring disorders, from birth until 2021, in relation to maternal T1DM stratified on cesarean section and/or preterm birth (CS/PTB, yes/no).

**Child disorder**	**Stratified by perinatal risk factors (CS/PTB)[a]**	**No endocrine AD N=1 076 751**				**T1DM N=5927**			
		N	HR	95% CI		N	HR	95% CI	
Any disorder (Any F)	No CS/PTB	176 892	1	1.00 (Reference)		378	**1.22**	1.10	1.35
	CS/PTB	50 305	**1.17**	1.16	1.19	1227	**1.51**	1.43	1.60
**Major psychiatric disorders combined**	No CS/PTB	83 742	1	1.00 (Reference)		166	**1.19**	1.03	1.39
	CS/PTB	21 127	**1.08**	1.07	1.10	469	**1.35**	1.23	1.48
Mood disorders*	No CS/PTB	47 673	1	1.00 (Reference)		100	**1.31**	1.08	1.59
	CS/PTB	11 707	**1.07**	1.05	1.09	259	**1.36**	1.21	1.54
Anxiety disorders*	No CS/PTB	61 202	1	1.00 (Reference)		120	**1.18**	0.98	1.41
	CS/PTB	15 533	**1.09**	1.07	1.11	357	**1.40**	1.27	1.56
**Neurodevelopmental disorders combined**	No CS/PTB	117 689	1	1.00 (Reference)		265	**1.25**	1.11	1.41
	CS/PTB	35 806	**1.23**	1.22	1.25	938	**1.64**	1.54	1.75
Intellectual disabilities	No CS/PTB	7299	1	1.00 (Reference)		23	**1.75**	1.16	2.64
	CS/PTB	3130	**1.75**	1.67	1.83	82	**2.34**	1.89	2.91
Specific developmental disorder (SDD)	No CS/PTB	52 813	1	1.00 (Reference)		130	**1.37**	1.15	1.63
	CS/PTB	17 872	**1.36**	1.34	1.38	510	**2.00**	1.84	2.19
Autism spectrum disorders (ASD)	No CS/PTB	12 825	1	1.00 (Reference)		30	**1.30**	0.91	1.86
	CS/PTB	4018	**1.18**	1.14	1.22	100	**1.52**	1.25	1.85
Behavioral and emotional disorders	No CS/PTB	85 180	1	1.00 (Reference)		187	**1.21**	1.05	1.4
	CS/PTB	24 411	**1.17**	1.16	1.19	640	**1.53**	1.41	1.65
ADHD	No CS/PTB	41 103	1	1.00 (Reference)		102	**1.36**	1.12	1.65
	CS/PTB	11 755	**1.17**	1.14	1.19	326	**1.58**	1.42	1.76
Emotional, social and tic disorders	No CS/PTB	32 522	1	1.00 (Reference)		70	**1.17**	0.93	1.48
	CS/PTB	9016	**1.13**	1.10	1.15	236	**1.45**	1.28	1.65
Other behavioural and emotional disorders	No CS/PTB	22 384	1	1.00 (Reference)		43	**1.04**	0.77	1.41
	CS/PTB	7029	**1.28**	1.24	1.31	206	**1.84**	1.61	2.12
Feeding disorders	No CS/PTB	3907	1	1.00 (Reference)		11	**1.45**	0.80	2.63
	CS/PTB	1818	**1.77**	1.67	1.87	55	**2.58**	1.98	3.37

Analysis of offspring born to mothers with Type 1 diabetes mellitus (T1DM), stratified by perinatal risk factors: cesarean section (CS) and/or preterm birth (PTB, before 37 weeks of gestation). Outcomes include psychiatric and neurodevelopmental disorders in offspring. The minimum age at onset of diagnosis was 15 years for personality disorders, 10 years for psychotic disorders, mood disorders, and eating disorders, and 5 years for anxiety disorders.

Any ICD-10 F diagnosis includes F00-F98. Major psychiatric disorders includes F20–F23, F25; F30–F34, F39; F40–F45; F50; F60, F63. Neurodevelopmental disorders (NDDs) includes F70–F73, F79; F80–F83; F84; F90–F91; F93–F95; F98.

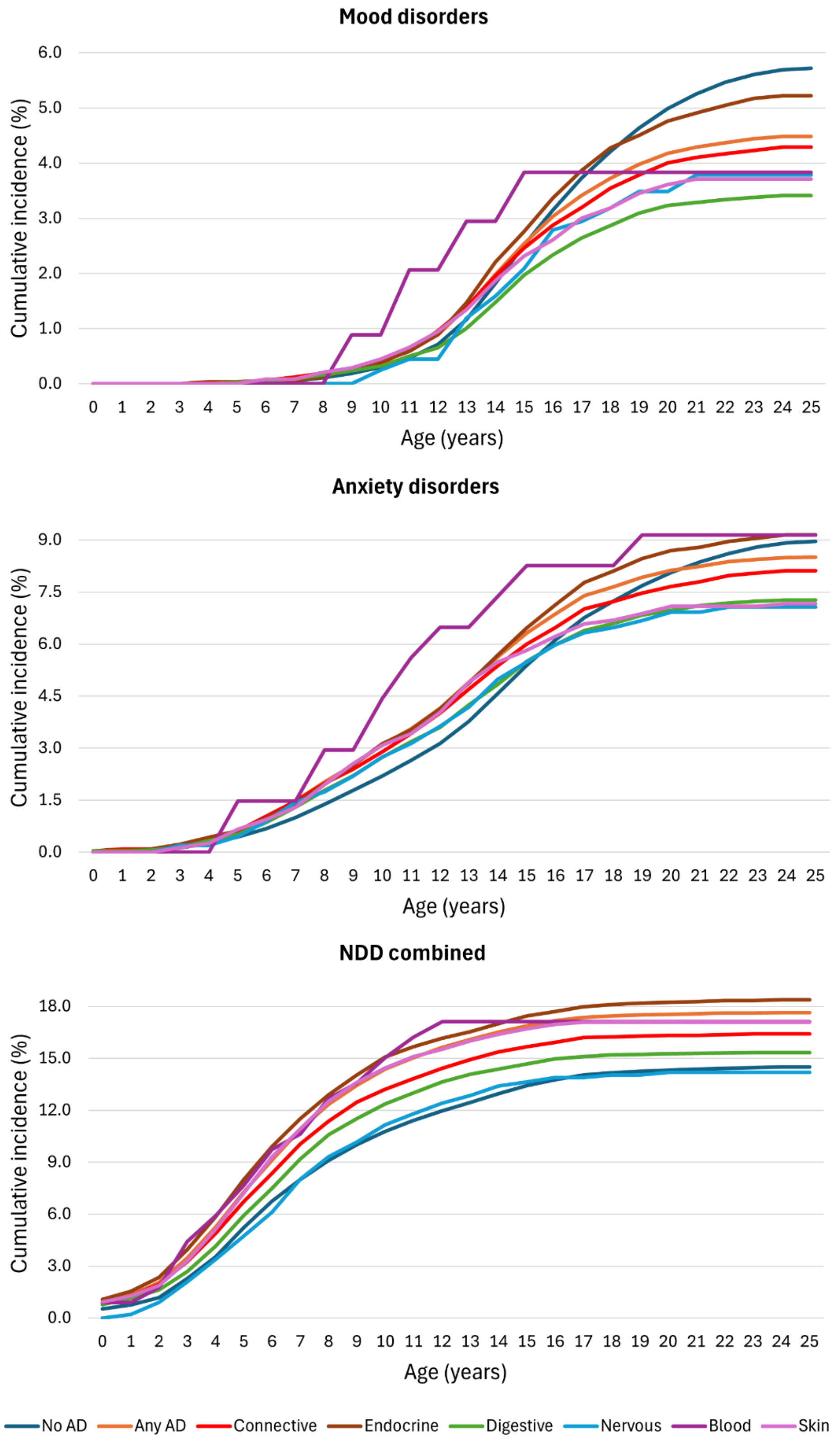

Supplementary Figure S1. Cumulative incidence of mood disorders, anxiety disorders and neurodevelopmental disorders (NDD) combined across age of the born children, stratified by exposure to different maternal autoimmune or autoinflammatory disorders (ADs) grouped by body system, and the group of children not exposed during pregnancy to any AD (dark blue).

References

Sankilampi U, Hannila ML, Saari A, Gissler M, Dunkel L. New population-based references for birth weight, length, and head circumference in singletons and twins from 23 to 43 gestation weeks. *Ann Med* 2013. 45, 446–454.

Clayton PE, Cianfarani S, Czernichow P, Johannsson G, Rapaport R, Rogol A. Management of the child born small for gestational age through to adulthood: a consensus statement of the International Societies of Pediatric Endocrinology and the Growth Hormone Research Society. *J Clin Endocrinol Metab* 2007. 92, 804–810.
